# Overexpression of native *Musa*-miR397 enhances plant biomass without compromising abiotic stress tolerance in banana

**DOI:** 10.1038/s41598-019-52858-3

**Published:** 2019-11-11

**Authors:** Prashanti Patel, Karuna Yadav, Ashish Kumar Srivastava, Penna Suprasanna, Thumballi Ramabhatta Ganapathi

**Affiliations:** 1 Plant Cell Culture Technology Section, Nuclear Agriculture and Biotechnology Division, Bhabha Atomic Research Centre, Trombay, Mumbai, India; 2 Plant Stress Physiology and Biotechnology Section, Nuclear Agriculture and Biotechnology Division, Bhabha Atomic Research Centre, Trombay, Mumbai, India; 3 Homi Bhabha National Institute, Mumbai, India

**Keywords:** Molecular engineering in plants, Transgenic plants

## Abstract

Plant micro RNAs (miRNAs) control growth, development and stress tolerance but are comparatively unexplored in banana, whose cultivation is threatened by abiotic stress and nutrient deficiencies. In this study, a native *Musa*-miR397 precursor harboring 11 copper-responsive GTAC motifs in its promoter element was identified from banana genome. *Musa*-miR397 was significantly upregulated (8–10) fold in banana roots and leaves under copper deficiency, correlating with expression of root copper deficiency marker genes such as *Musa-*COPT and *Musa-*FRO2. Correspondingly, target laccases were significantly downregulated (>−2 fold), indicating miRNA-mediated silencing for Cu salvaging. No significant expression changes in the miR397-laccase module were observed under iron stress. *Musa-*miR397 was also significantly upregulated (>2 fold) under ABA, MV and heat treatments but downregulated under NaCl stress, indicating universal stress-responsiveness. Further, *Musa-*miR397 overexpression in banana significantly increased plant growth by 2–3 fold compared with wild-type but did not compromise tolerance towards Cu deficiency and NaCl stress. RNA-seq of transgenic and wild type plants revealed modulation in expression of 71 genes related to diverse aspects of growth and development, collectively promoting enhanced biomass. Summing up, our results not only portray *Musa*-miR397 as a candidate for enhancing plant biomass but also highlight it at the crossroads of growth-defense trade-offs.

## Introduction

MicroRNAs (miRNAs) are 20–24 nucleotide (nt) non-coding RNAs, which silence downstream target genes in plants and animals. They are generated through a complex processing pathway starting with RNA Pol II mediated-transcription of a long primary microRNA (pri-miRNA) from genomic locus. A complex of nuclear DICER/DCL RNase and RNA binding proteins HYPONASTIC LEAVES (HYL1) and SERRATE (SE) processes pri-miRNA into shorter stem-looped precursor miRNA (pre-miRNA). This is further processed into mature 20–24 nt duplex miRNA consisting of guide (miRNA) and passenger (miRNA*) strands. Following 3’ 2-O-methylation by HUA ENHANCER (HEN1), the stabilized duplex is loaded onto ARGONAUTE (AGO) protein as part of the miRNA-induced silencing complex (miRISC), which is exported into cytosol to recognize the cognate target mRNA. Subsequently, the guide miRNA strand either mediates mRNA cleavage via AGO or induces translational inhibition. Thus, plant miRNAs control gene expression networks and orchestrate multiple processes including growth, development and abiotic/biotic stress tolerance^1^.

Plant adaptation under stress is energy and resource-expensive, resulting in trade-offs against growth and biomass increase, which negatively impact crop productivity^2^. Banana (*Musa* spp) is one such fruit crop, important for economically backward regions worldwide, especially in India where the whole plant is culturally and commercially important. The fruit is nutritious, energy dense and palatable to infants^3^. Worldwide banana cultivation amounted to 114 million tonnes in 2016–2017 of which India topped the list with approximately 30 million tonnes^4^. However, banana cultivation undergoes severe production losses when challenged with stress^5,6^, thus warranting improvement measures. At present, tissue culture and genetic engineering (GE) for tolerance-conferring traits are feasible options^7^. Of the two however, GE through transgenic technology has proved highly promising. Various stress-related genes such as dehydrins, WRKYs, aquaporins, Stress-Associated Proteins (SAPs) and ferritin were found to successfully protect the plant from abiotic stresses^5,8^. In this regard, the field of miRNA research has remained relatively unexplored with respect to its potential application in crop improvement though miRNAs are critical to the plant developmental programme and stress response^9^. Genome-wide studies on the miRNA expression profiles of banana under different stresses have only recently been initiated^10–12^. However, with the exception of miR156 known to control structure and development^13^ to the best of our knowledge, no other miRNAs have been functionally characterized in banana till date. Detailed studies of other miRNAs are necessary to elucidate aspects of crop physiology that may be important for downstream improvement measures.

Micronutrient deficiency as a class of abiotic stress, severely impacts plant growth and development because these elements are essential cofactors in enzymatic reactions. Particularly iron (Fe) and copper (Cu) are redox-active cofactors in photosynthesis, respiration and antioxidant enzymes, rendering them important for normal plant development^14^. Copper is also required for lignin polymerization and circadian clock control^15,16^. Thus, its deficiency retards growth, disrupts photosynthesis, cell wall metabolism, hormone and antioxidant activity, resulting in chlorotic young leaves with curling margins and stunted appearance. In fruit crops like banana, even mild asymptomatic Cu deficiency due to excessive soil nitrogen, can heavily impact yield^17^. Imminent Cu deficiency may often be overlooked, until progression to severe stages with irrevocable damage^18,19^. Copper is also a potent generator of reactive oxygen species (ROS) and contributes to damage by other abiotic stresses. Further, it exhibits cross talk with Fe in plants as both have substitutable one-electron transfer ability and under Fe deficiency, an increased uptake of Cu compensates for decreased activity of Fe-containing enzymes^16,20^. Plants have thus evolved a sophisticated system of Cu uptake transport and chelation, which is controlled in part by the master transcriptional factor (TF) SQUAMOSA PROMOTER BINDING-LIKE 7 (SPL7). Due to the obligate requirement of Cu by the photosynthetic enzyme plastocyanin (PC), this system also deploys the four special SPL7-regulated “Cu-miRNAs” namely miR397, miR398, miR408 and miR857, to downregulate dispensable Cu proteins and divert the limiting metal to PC^21^. Of these miRNAs, miR397 presents a particularly interesting case, as a highly conserved miRNA that targets selected laccase family members in *Arabidopsis* and rice through transcript cleavage^22^. Laccases are multicopper oxidases (MCOs) using molecular oxygen and the one-electron transfer ability of Cu to oxidize diverse phenolic substrates, such as monolignols in the lignin biosynthesis pathway^23^. Thus, they are essential to cell wall and vascular integrity maintenance, which implies their roles in defense against stress^24,25^. MicroRNA 397 is also reported to significantly influence plant growth and yield in *Arabidopsis* and rice^26,27^. Furthermore, miR397 is stress responsive^28^ raising the possibility that it integrates Cu homeostasis and stress tolerance with overall plant architecture.

With this background, the present study was done to functionally characterize native *Musa-*miR397 in the context of Cu deficiency response in banana. Expression profiling of *Musa-*miR397 revealed upregulation under Cu deficiency, abscisic acid (ABA), heat and methyl viologen (MV) treatment and repression under salinity stress. This was simultaneous with repressed expression of target laccases, specifically under Cu deficiency. The miR397 overexpressing transgenic lines showed enhanced biomass, without compromising abiotic stress tolerance. Furthermore, *Musa*-miR397 overexpression led to alteration of genes associated with plant growth and development, thus possibly contributing to increased growth vigor.

## Results and Discussion

### Molecular analysis in wild-type banana under copper and iron deficient conditions

#### Expression analysis of root transporters under Cu deficiency in banana

**Figure 1:**
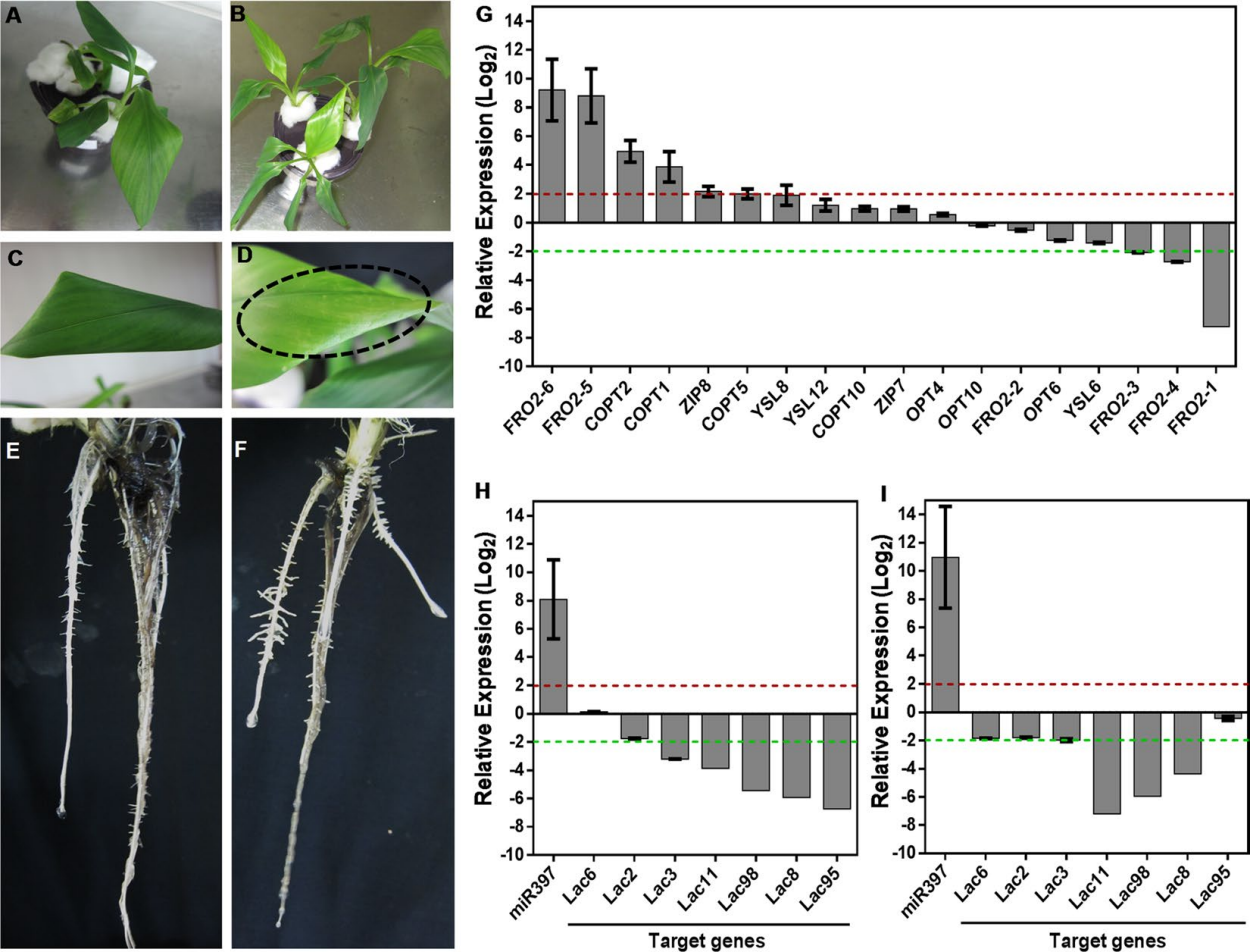
Molecular basis of copper deficiency response in banana. Healthy hydroponically grown banana plantlets were challenged with copper deficient conditions (0 µM copper +200 µM BCS). Plants growing in half-strength MS with 50 nM copper were taken as controls. (**A**,**B**) Plants growing in control and copper deficient conditions respectively. (**C**,**D**) Insets of (**A**,**B**) showing leaf from control and copper deficient plants respectively. The dotted circle highlights whitish spots on the copper deficient leaf. (**E**,**F**) Roots from control and copper deficient plants respectively. (**G**) Expression profiling of copper-responsive transporters belonging to the *Musa-FRO*, *Musa-COPT*, *Musa-OPT*, *Musa-YSL* and *Musa-ZIP* families in roots of copper deficient plants. (H-I) Expression profiling of *Musa*-miR397 and target laccases (*Musa-Lac6*, *Musa-Lac2*, *Musa-Lac3*, *Musa-Lac11*, *Musa-Lac98*, *Musa-Lac8*, *Musa-Lac95*) in root (**H**) and leaf (**I**) of copper deficient plants. Bars are mean of 3 replicates ± SE and represent log_2_ fold expression of the miRNA/target over control condition. Gene expression was normalized to that of *Musa-EFα*. Baseline represents expression of the genes in the control condition. Dotted lines indicate fold change cut-off of 2.

Wild-type banana plants showed distinct phenotypic differences under Cu deficient conditions in terms of whitish spots on youngest newly emergent leaf (Fig. 1A–D). The Cu deficiency also altered root architecture (RA) with more lateral root (LR) emergence from the main cord roots compared with that of control (Fig. 1E,F). Our observations agree with the finding in *Arabidopsis* that Cu deficiency increases lateral root density without affecting primary and lateral root length, reflecting enhanced scavenging for residual copper^29^. Since the major effect was seen on roots, various transporters were selected and their expression was measured in roots. These included Cu-responsive marker genes, such as *FRO* family of ferric chelate reductases, *COPT* family of high-affinity copper transporters, metal transporters *ZRT-IRT- like* (*ZIP*), *OLIGOPEPTIDE TRANSPORTER* (*OPT*) and metal-*Nicotiana*mine (NA) transporters of the *yellow stripe-like* (*YSL*) family^30^. Applying 2 fold cut-off allowed us to differentiate genes with significant up- or down-regulation under Cu deficiency (Fig. 1G). Strikingly five of the six members of *FRO2* family in banana were among the top-ranked genes altered under Cu deficiency. Of these, *Musa-FRO2–5* and *Musa-FRO2-6* were maximally up regulated by 8.8 and 9.2 fold, respectively; while *Musa-FRO2-1* was downregulated by −7.21 fold followed by *Musa-FRO2-3* (−2 fold) and *Musa-FRO2-4* (−2.72 fold). Members of the FRO family reduce Fe^+3^ and Cu^+2^ ions at the plasmalemma and hence are known to be upregulated under metal-deficient conditions. For instance, *Arabidopsis FRO2-4* and *FRO2-5* are upregulated in Cu deficient roots^31,32^. The differential regulation of *Musa-FRO2* family under Cu deficiency in roots reflects the plant requirement for Cu, with likely concomitant prevention of unnecessary and potentially toxic Fe uptake through the repression of *Musa-FRO2-1, Musa-FRO2-2 and Musa-FRO2-3*. Besides FRO, *Musa-COPT* family members were also induced which mediate high affinity Cu uptake in a spatially and temporally regulated manner with differing sensitivities to Cu deprivation^33,34^. The maximum induction was observed in three members: *Musa-COPT2* (4.94 fold) followed by *Musa-COPT1* (3.87 fold) and *Musa-COPT5* (1.99 fold). The induction of *Musa-COPT* genes in banana roots depicts the onset of Cu deficiency in the plant, with *Musa-COPT2* and *Musa-COPT1* being the most responsive to Cu limitation in the roots; while *Musa-COPT10* transcription may not be regulated by Cu status, or alternatively may have a different spatial/temporal expression altogether. Of the two *Musa-ZIP* genes, *Musa-ZIP8* was upregulated by 2.16 fold, highlighting its probable role as a Cu ion membrane transporter like that of *Arabidopsis* ZIP2^35^. Among other gene families, *Musa-OPT4, Musa-OPT6 and Musa-OPT10* genes as well as *Musa-YSL6, Musa-YSL8 and Musa-YSL12* genes showed no significant alteration. *Musa-OPT6* and *Musa-YSL6* were repressed by −1.23 and −1.41 folds respectively, while *Musa-YSL8* and *Musa-YSL12* were upregulated by 1.89 and 1.2 folds respectively. Though *At*OPT3 transports Cu^36^, OPT genes are principally involved in systemic Fe signaling^37,38^, which may explain the downregulation of *MusaOPT6* and negligible alteration in *Musa-OPT4/10* under Cu deficiency. The differential regulation of the YSL genes indicated an effort to maintain flux of other micronutrients like Fe that could be affected by Cu uptake as well as transporting Cu-NA chelates^39,40^. Taken together, our results point towards a conserved system of Cu sensing and increased Cu acquisition by the banana root system under Cu deficient conditions.

#### Musa-miR397and laccase expression under Cu deficiency in banana

As described earlier, Cu deficiency response is also dependent on miRNA-mediated regulation. We therefore analyzed the expression of native *Musa-*miR397 and the seven *Musa*-*Lac* targets in roots and leaves of banana under Cu deficiency. Preliminary analysis of a 2500 nt sequence upstream of *Musa*-miR397 revealed 11 copper-responsive GTAC *cis*-elements within the proximal 500 nt region (Supplementary Fig. S1) indicating involvement of *Musa*-miR397 in Cu deficiency. In the *Arabidopsis* Cu deficiency response SPL7 binds to conserved GTAC sequences in the core promoters of Cu responsive genes such as *COPT1/2*, *ZIP*, *YSL2*, *FeSOD* and activates transcription^30,32,41^. This motif is also overrepresented in the *At*miR397 promoter, which is strongly activated by SPL7 under Cu deficiency^18,42^. Applying 2 fold cut-off, *Musa-*miR397 was upregulated in both roots and leaves (8.08 and 10.93 folds respectively) under Cu deficiency with concomitant downregulation of targets in both tissues (Fig. 1H–I), though more consistently in roots compared to leaves. In both root and leaf, *Musa*-*Lac8*, *Musa*-*Lac98* and *Musa*-*Lac11* were maximally downregulated by more than 3 fold, indicating them as dominant targets for miR397 under copper deficiency. In addition, organ-specific targets were also observed. For instance, *Musa*-*Lac95* displayed strong downregulation in root (−6.74 fold) but remained unaltered in leaf. Conversely, *Musa*-*Lac6* was unaltered in root but was downregulated in leaf (−1.85 fold). Our results thus prove that *Musa-*miR397 expression is highly sensitive to mild Cu deficiency and it can target different laccases in both root and shoot. Analysis of *Musa*-*Lac* protein sequences showed remarkable conservation of four predicted Cu binding domains (CuBD), a proline-rich C terminal and other amino acid motifs, when compared with *Arabidopsis* laccases. Furthermore, these laccases were predicted to contain a non-conserved N-terminal signal peptide, implying possible secretion into extracellular space or into the cytosol from intracellular organelles (Supplementary Fig. S6)^43^. These features suggest their likely roles as at least partial Cu reserves^44^. Thus, in line with the Cu-economy model of regulation for *Arabidopsis*^21^, we postulate that under Cu deficiency, activation of *Musa-*miR397 may silence the laccase targets by transcript cleavage and salvage available metal for photosynthetic and other essential functions. Integration of the response of other Cu deficiency associated marker genes with miR397 activity allows banana to enhance the root uptake of copper and its redistribution to organs, while minimizing toxicity associated with non-specific metal uptake.

#### Musa-miR397 and laccase expression under Fe deficiency and excess in banana

Considering that Cu deficiency induces secondary Fe deficiency in plants^20,32^ and that both metals share transporters of the *FRO*, *COPT*, *ZIP*, *OPT* and *YSL* families^45^, we investigated the expression of *Musa-*miR397-laccase module under Fe deficiency and excess, with a 1 fold cut-off in expression values. In contrast to the significant upregulation under Cu deficiency, miR397 was not significantly altered by Fe starvation but downregulated (−1.25 fold) by excess Fe treatment (400 μM) for 3 days. Expression changes in the target laccases were below the cut-off and a weak reciprocal regulation with the miRNA was observed (Supplementary Fig. S2). We also tested *Musa-COPT2* in both Fe deficiency and excess conditions, as its *Arabidopsis* counterpart is almost 6-fold induced by Fe deficiency in roots for enhanced Cu uptake^19,32^. However, *Musa-COPT2* expression was not altered in either condition, in contrast with its significant induction under Cu deficiency. These results suggested that the plants were probably undergoing an early Fe deficiency response without disturbance of Cu status and therefore had not yet increased the compensatory uptake of Cu. In case of Fe excess, miR397 was downregulated and coupled with the negligible alteration in *Musa-COPT2*, may again reflect early response to Fe excess, without change in Cu uptake. Indeed, no significant change was observed in Cu content of rice leaves exposed to Fe toxicity over 4 days^46^. Taken together, our results suggest that though Cu deficiency response and Fe homeostasis in banana share many of the known Fe-regulated transporters, Cu specifically regulates the *Musa*-miR397-laccase module, at least in the root tissue.

### Expression profiling of *musa-*mir397–laccase module under different abiotic stresses

**Figure 2:**
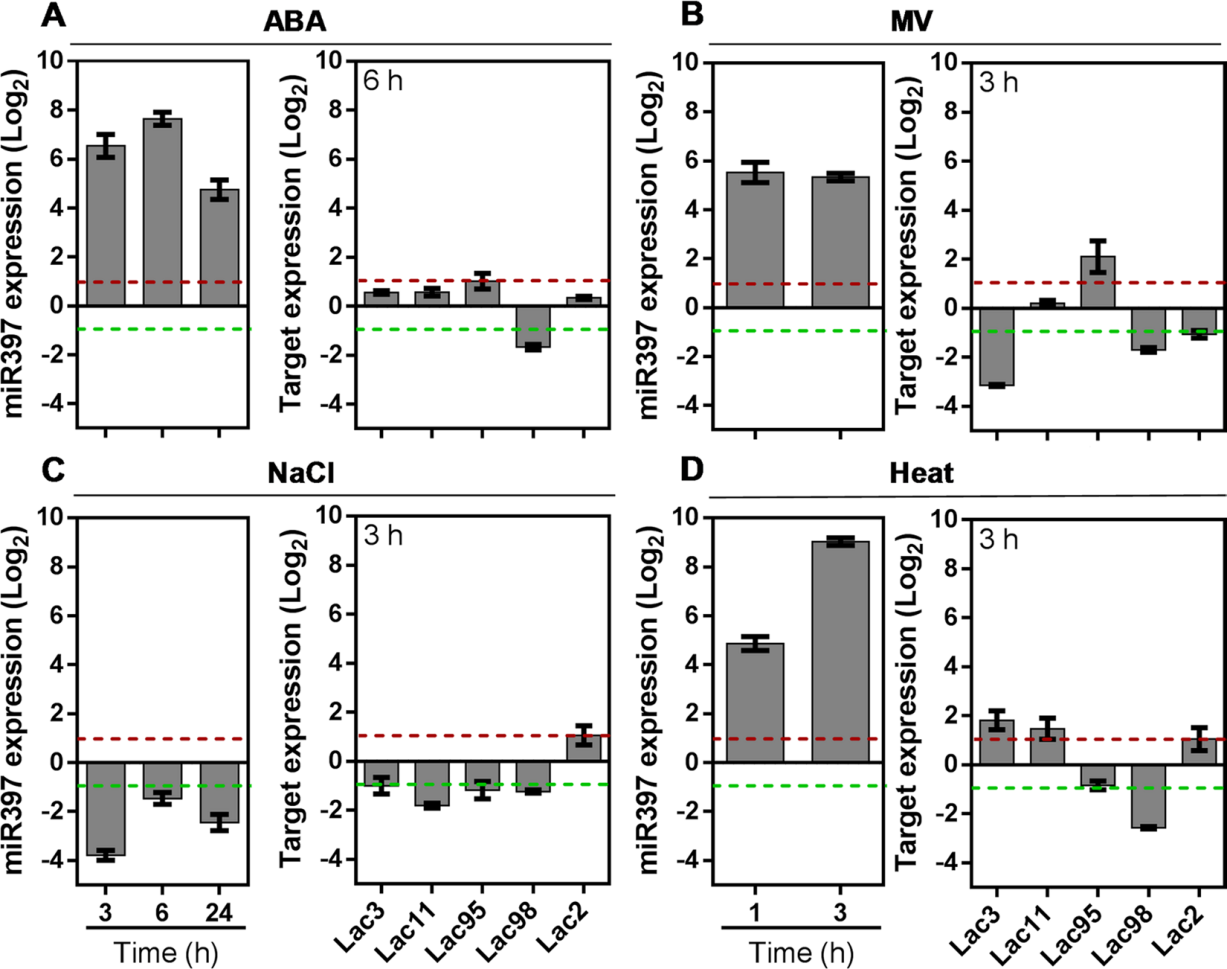
Expression profiling of *Musa*-miR397-laccase module under various abiotic stresses. Healthy hydroponically grown banana plantlets were exposed to ABA, MV, heat and salt stresses for the indicated time periods to ascertain the expression pattern of the pri-Musa-miR397 and target laccases *Musa-Lac3*, *Musa-Lac11*, *Musa-Lac95*, *Musa-Lac98* and *Musa-Lac2* (**A**–**D**). *Musa*-miR397 expression was analyzed under (**A**) 100 µM ABA treatment for 3, 6 and 24 h (**B**) 100 µM MV stress for 1 and 3 h (**C**) 90 mM NaCl stress for 3, 6 and 24 h (**D**) heat stress at 45 °C for 1 and 3 h. The target laccase genes were analyzed at the time point of maximum pri-miR937 expression change (6 h of ABA treatment, and 3 h each for MV, NaCl and heat treatments). Bars are mean of 3 replicates ± SE and represent log_2_ fold expression of the miRNA/target over control condition. Gene expression was normalized to that of *Musa-EFα*. Baseline represents expression of the genes in the control condition. Dotted lines indicate fold change cut-off of 1.

Analysis of the upstream sequence of *Musa*-miR397 yielded stress-related motifs, such as the ABA-responsive (ABRE), dehydration responsive (MYC, CBF) and hormone responsive (salicylic acid and jasmonic acid) elements (Supplementary Fig. S1). Similar elements were also observed in the promoters of miR397 gene from food crop grass species and *Arabidopsis*, indicating possible involvement of this miRNA in stress response^47^. We thus evaluated the expression of pri-*Musa*-miR397 under different treatments. Under ABA treatment, *Musa-*miR397 was upregulated by 6.53, 7.64 and 4.75 fold at 3, 6 and 24 h treatment duration, respectively. Exposure to MV resulted in upregulation of *Musa-*miR397 by more than 5 fold at 1 and 3 h after treatment. In case of heat, *Musa-*miR397 was upregulated by 4.86 and 9.02 fold at 1 and 3 h, respectively. Unlike ABA, heat and MV, the exposure of salt stress for 3, 6 and 24 h resulted in downregulation of *Musa*-miR397 level by 3.79, 1.46 and 2.45 fold, respectively (Fig. 2A–D). Our results thus highlight the generalized stress-responsive character of *Musa-*miR397 as also reported in wheat^28^ rice^48^, radish^49^, soybean^50^ and citrus^51^. In heat-stressed banana fruit, miR397 was upregulated^12^, validating our findings. Similarly, salt-mediated downregulation of miR397 was reported in banana^52^ and sugarcane^53^. In line with the basic function of *Musa*-miR397 in Cu homeostasis, therefore, we propose two theories to explain the significant upregulation of *Musa*-miR397 under ABA, heat and MV treatments. Firstly, modulation of *Musa*-miR397 under stress may have an underlying ROS component, as ROS generation is common to abiotic stresses and miR397 is upregulated by ROS in rice^54^. Copper itself indirectly contributes to ROS metabolism^55,56^ and also modulates plant development^15^. Hence disruption of plant growth under stress can impact cellular Cu homeostasis, resulting in activation/repression of miR397-mediated silencing^21^. For instance, salt stress reduces plant photosynthetic rate^57^. Considering an equivalent situation in our salt-treated banana plants therefore, the demand for Cu may decrease, resulting in the observed feedback downregulation of *Musa*-miR397. Secondly, involvement of the ABA signaling pathway, which is activated under different abiotic stresses, can also be considered. Here too, ABA signaling depends on Cu homeostasis, because ABA-mediated stomatal closure requires a Cu-amine oxidase^58–61^. In light of our *cis*-element analysis, we therefore propose that ABA signaling under the different stresses applied, may alter downstream *Musa*-miR397 expression, likely through the ABRE element in the putative promoter region. Taken together, the present study reports the involvement of *Musa*-miR397 in different stresses, through likely global effects on Cu homeostasis and ROS signaling, which remain to be investigated.

Towards elucidating the strength of the *Musa*-miR397-target module under stress, we also analyzed laccase expression, at the time point of maximum miRNA alteration in each stress condition. Appling 1 fold cut off, among the targets, only *Musa*-*Lac98* was uniformly downregulated by −1.67, −1.7, −2.56 and −1.24 folds under ABA, MV, heat and NaCl treatments, respectively. Other laccases were differentially regulated. At 6 h ABA treatment, *Musa*-*Lac95* was upregulated by 1.02 fold, while expression of *Musa*-*Lac3*, *Musa*-*Lac11* and *Musa*-*Lac2* remained unchanged. Exposure to MV for 3 h led to downregulation of *Musa*-*Lac3* and *Musa*-*Lac2* by −3.15 and −1.06 folds respectively, while *Musa*-*Lac95* was upregulated by 2.1 fold and *Musa*-*Lac11* had no significant change in expression. At 3 h heat stress, *Musa*-*Lac3*, *Musa*-*Lac11* and *Musa*-*Lac2* were upregulated by 1.81, 1.47 and 1.04 folds respectively, while *Musa*-*Lac95* was not significantly altered. However, under salt stress at 3 h, *Musa*-*Lac3*, *Musa*-*Lac11*, *Musa*-*Lac95* were downregulated by −1.0, −1.82 and −1.18 folds respectively; while *Musa*-*Lac2* was upregulated by 1.05 fold (Fig. 2A–D). We thus did not obtain uniform reciprocal regulation of laccase targets with *Musa*-miR397 under different stress conditions. This may occur due to uncoupling of miRNA-target relationship by independent factors regulating individual laccase transcription or changes in protein stability, which may not reflect at transcript level. Alternatively, other laccases, which are not *Musa*-miR397 targets, may be predominantly operational under these stresses. Stress-imposed modulation of the proteins catalyzing processing steps between pri- and mature miRNAs, may also add complexity to the *Musa*-miR397-laccase module^62^. Specifically, under salt stress in our study, four target laccases were repressed in banana leaf, different from a previous report in sugarcane roots^53^. This may reflect tissue-specific regulation of the same laccase gene. Additionally, under salt stress, banana miR397 was predicted to target a protein kinase, which increases the repertoire of targets under stress^52^. The present study thus supports the broad involvement of the *Musa*-miR397-laccase module in diverse stresses, and depicts a complex non-straightforward regulation of targets under stress, subject to further validation.

### Overexpression of *musa*-mir397 enhances plant biomass in banana

Bioinformatics analysis of putative *Musa*-miR397 primary sequence revealed a typical stem-loop structure with 3 A-C mismatches between the miRNA: miRNA* (Supplementary Fig. S3) and the conserved nature of the 21-nt mature form (Supplementary Fig. S3), indicating that the genomic locus codes for a genuine miRNA. We obtained a 6.4–7.0 fold increase in the mature miRNA in six lines analyzed over WT, respectively. Three lines named *Musa*-miR397Ox-1, *Musa*-miR397Ox-2 and *Musa*-miR397Ox-3 were chosen for further experiments based on significant expression, profuse clonal shoot production and single-copy number in Southern blotting (Supplementary Fig. S3). Further, we analyzed the expression pattern of the target laccases in line *Musa*-miR397Ox-3 (Supplementary Fig. S3). Relative to WT, targets *Musa*-*Lac3*, *Musa*-*Lac11*, *Musa*-*Lac95*, *Musa*-*Lac6*, *Musa*-*Lac8*, *Musa*-*Lac98* and *Musa*-*Lac2* were significantly repressed by −4.44, −2.68, −1.68, −2.73, −2.57, −3.72 and −2.64 folds, respectively. The miR397-laccase module is reported to influence cell wall lignification in *Arabidopsis* and poplar^22,27^. Therefore, we stained transgenic and WT roots with toluidine blue O^63^ to determine the extent of lignin deposition as bluish-green coloration around the vasculature. As expected, miR397Ox-3 roots displayed less intense staining compared with WT (Supplementary Fig. S4). This could be attributed to *Musa*-miR397-mediated repression of target laccases (Fig. 1B,C and Supplementary Fig. S3) as laccases catalyze monolignol polymerization into lignin, and are predicted to be apoplastic in localization^23^ (Supplementary Fig. S6). Together, the data confirmed that overexpression of *Musa*-miR397 downregulated the target laccases leading to reduced vascular lignification.

**Figure 3:**
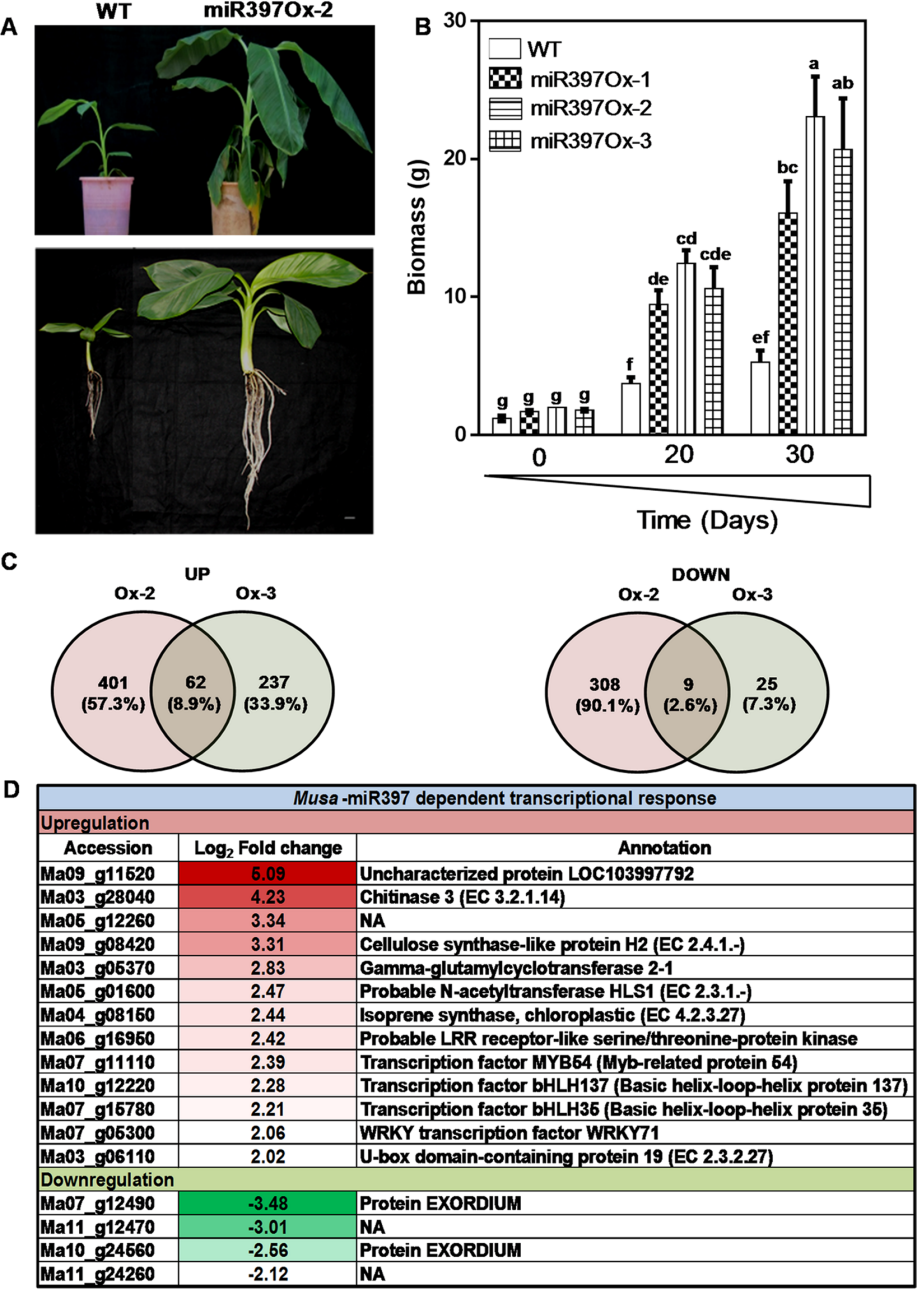
Overexpression of *Musa*-miR397 enhances plant biomass in banana. Healthy wild-type (WT) plants and transgenic lines *Musa-*miR397Ox-1*, Musa-*miR397Ox-2*, Musa-*miR397Ox-3 were evaluated for growth performance under control conditions. (**A**-upper panel) Greenhouse hardened WT and transgenic line *Musa-*miR397Ox-2 aged 4 mo. (**A**-lower panel) Hydroponically grown WT and transgenic line *Musa*-miR397Ox-2 aged 2 mo. (**B**) Measurement of biomass (as fresh weight) of WT and transgenic lines over 20 and 30 d in hydroponics. Bars represent the mean value of atleast 3 replicate plants ± SE. Means were compared using one-way ANOVA and Duncan’s multiple range test. Small alphabets represent statistically significant differences in the means. (**C**) RNA-sequencing of *Musa*-miR397Ox-2, *Musa*-miR397Ox-3 and WT plants. Differentially expressed genes (DEGs) between each line and the WT were obtained after applying adjusted *p*-value cut-off <0.05. Venn diagrams depict the distribution of up- and down-regulated DEGs common between and exclusive for each line. (**D**) Selected up- and down-regulated genes which are common to both transgenic lines, obtained after applying 2 fold cut-off in expression values. Accession numbers were derived from the Banana Genome Hub (https://banana-genome-hub.southgreen.fr). For each gene, annotation was assigned based on the homology search against viridiplantae protein sequences from Uniprot. Reference sequences were matched against Uniprot data using BLAST program. *NA*: not annotated.

Although both transgenic and wild-type banana plants were raised in identical media and hormone treatments, *Musa*-miR397Ox lines had significantly increased vegetative growth, as measured by biomass increase, than WT plants at two months in hydroponics and four months hardened in the greenhouse conditions (Fig. 3A top and bottom panels). *Musa*-miR397Ox-1, *Musa*-miR397Ox-2 and *Musa*-miR397Ox-3 showed an increase of 1.95, 2.57 and 2.19 folds in fresh weight respectively, over wild-type at 20 days and 2.05, 2.94 and 2.63 folds respectively over wild-type at 30 days (Fig. 3B). Since enhanced micronutrient uptake positively correlates to plant biomass and yield^64^, we analyzed the micronutrient profile of miR397Ox lines and wild-type plants. However, transgenic lines did not show any significant trend for Fe, zinc (Zn), Cu and manganese (Mn) levels when compared with wild-type (Supplementary Fig. S3) indicating that overexpression of miR397 did not significantly perturb micronutrient uptake. We next performed leaf RNA-sequencing of *Musa*-miR397Ox-2 and *Musa*-miR397Ox-3 along with wild-type banana plants to gain insight into the molecular basis for the growth phenotype observed. Totally, 463 genes were upregulated and 317 genes downregulated between *Musa*-miR397Ox-2 and wild type banana, while 299 genes were upregulated and 34 genes downregulated between *Musa*-miR397Ox-3 and wild type banana. Of these, 62 genes were upregulated and 9 genes downregulated in common between both transgenic lines versus wild type (Fig. 3C, Supplementary Files F1 and F2). Applying a 2 fold cut-off for both categories, we obtained top-ranked genes which maximally contributed to the enhanced growth phenotype of miR397-Ox lines (Fig. 3D). Most of these genes encoded enzymes, structural proteins and TFs spanning diverse metabolic and structural aspects of plant growth and development.

The wall-modifying enzymes chitinase (Ma03_g28040), cellulose synthase-like (Ma09_g08420) and the TF MYB54 (Ma07_g11110), were induced >2 fold. Chitinases promote plant growth and development by modulating lignin deposition, cell shape, cellulose biosynthesis and root growth^65^. Overexpression of maize class I chitinase in tobacco enhanced plant growth^66^. Cellulose synthase- like genes (CSLs) catalyze polymerization of essential non-cellulosic wall polysaccharides and dynamically modify the matrix in response to environmental and internal growth signals^67^. Deposition of the secondary cell wall (SCW) is also part of the growth process and is promoted by the TF MYB54^68^.Thus upregulation of these genes in miR397Ox lines signifies extensive cell wall remodeling in accordance with increased plant growth. This was further corroborated through hormone analysis of transgenic and WT leaves, which revealed significantly reduced levels of the cytokinin (CK) trans-zeatin (tZ) in both *Musa*-miR397Ox-2 (80% reduction) and *Musa*-miR397Ox-3 (49% reduction), compared with WT (Supplementary Fig. S4). Trans-zeatin is known to positively influence lignification by a yet unknown mechanism, as decrease in shoot tZ levels led to lower lignin content in xylary tissue, which could be rescued by exogenous tZ application^69^. Also, overexpression of miR397 in pear caused significant reduction of seed lignin content, which coincided with decreased cytokinin levels^70^. In our study, the reduced content of tZ in *Musa*-miR397Ox plants coincides well with lower lignin deposition in these lines, though at present the exact relationship between the miR397-laccase module and tZ is unclear. However, our RNA-seq data revealed upregulation of an F-box/kelch repeat protein-coding gene (Ma07_g13140) in both transgenic lines compared with WT. This protein belongs to the KISS ME DEADLY (KMD) family forming an SCF complex of E3 ubiquitin ligases and targets type B-ARR transcription factors, which mediate the CK response in plants^71,72^, thus negatively regulating CK signaling. This family also negatively modulates the post-transcriptional activation of the phenylpropanoid pathway to external/internal stimuli, integrating the formation of lignin, bioactive phenolics, tannins and other secondary aromatics, with the plant requirement during growth especially in response to the carbon/nitrogen ratio^73^. Thus, *Musa*-miR397-mediated lowering of lignin deposition could be associated with altered CK signaling and warrants further investigation.

Nutrient status sensing and the ability to reallocate/recycle resources are critical to plant growth and development. In this context, sulfur (S) is important for both redox activity and protein synthesis. In our study, the upregulation of S-limitation induced-genes γ-glutamylcyclotransferase (*GGCT*, Ma03_g05370) and an APS sulfotransferase (*APR*, Ma02_g15730) probably exemplify adjusted S metabolism in *Musa*-miR397Ox plants. While GGCT recycles glutathione, APR is required for assimilation of S into cysteine^74,75^. Induction of both genes in miR397Ox plants may reflect increased S salvaging to balance normal redox homeostasis with biomass production, as would be expected if growth exceeds environmental S reserves.

We also observed increased expression of banana homologs of the genes *HLS1*, *ISPS* and *bHLH137* involved in hormone signaling, tissue/organ development and coordination of growth with light response. *HLS1 (*HOOKLESS1)/CONSTITUTIVE PHOTOMORPHOGENIC 3 (Ma05_g01600) encodes an N-acetyltransferase required for auxin signaling, unidirectional growth, photo- morphogenesis and ethylene response. Induction of *HLS1* in miR397Ox lines may result from growth-induced mechanical stress, releasing ethylene, which activates HLS1^76^. Contrastingly, *Arabidopsis* TF bHLH137 represses GA signaling and is inhibited by ERF11 to allow GA-mediated growth^77,78^. As different plant bHLH members positively or negatively influence cell size and biomass^79^, upregulation of banana *bHLH137* (Ma10_g12220) indicates complex cross talk between ethylene and GA in the miR397Ox plants. The ISOPRENE SYNTHASE (ISPS) enzyme synthesizes isoprene (IS) from dimethylallyl diphosphate in certain plant species. Notwithstanding high synthesis costs however, IS facilitates plant growth and defense, as overexpression of *ISPS* in *Arabidopsis* promoted overall plant growth and protection against photoinhibition. Isoprene also altered expression of phenylpropanoid pathway genes, the photosynthetic light responsive machinery, cell wall synthesis and those associated with stress responsiveness, hormone signaling, seedling germination and growth^80^, indicative of global transcriptomic effects. Thus enhanced expression of ISPS (Ma04_g08150) in miR397Ox banana plants may contribute to growth vigor. Additionally, several of the 62 upregulated genes were associated with primary wall modification and wall-to-cytoplasm signaling, carbon/nitrogen balance sensing, jasmonate signaling and organ/tissue development (Supplementary File F1). These genes may function in an auxiliary or coordinated manner with the above-discussed genes to integrate environmental and internal cues for growth enhancement.

Among the nine-downregulated genes, two *EXORDIUM* (*EXO*) genes (Ma07_g12490 and Ma10_g24560) were significantly repressed (−2.5 to −3.4 fold). The EXO protein promotes brassinosteroid (BR)-mediated shoot vegetative growth through control of wall-modifying genes leading to cell wall expansion^81,82^. A related gene *EXORDIUM LIKE-1* is also required for growth under C-limiting conditions^83^ and *exo* mutants have impaired sugar sensing^84^. In the present study it is not clear why *EXO* was downregulated in *Musa*-miR397Ox lines, though it may result from altered BR signaling in the transgenic lines. Brassinosteroids modulate plant growth in diverse ways, in a feedback-regulated manner^85–90^. We found changes in BR biosynthesis, signaling and downstream effector genes, in each of the *Musa*-miR397Ox lines relative to wild type (Supplementary File F2). Another gene *TEMPRANILLO 2* (*TEM2*), a senescence-promoting ethylene-responsive transcriptional repressor, was also downregulated. It is itself repressed in young flowers to prevent premature senescence^91^. In long-day flowering *Arabidopsis*, TEM1 and TEM2 repress flowering induction genes to correctly time the vegetative-to-floral transition^92,93^. Therefore, though *Musa* spp is a day-neutral flowering herb, the repression of *TEM2* in miR397Ox banana plants may reflect accelerated maturation of the transgenics relative to WT. Thus altogether, the data suggested substantial reprogramming of gene expression to achieve increased growth vigor of miR397Ox plants. However a direct role for the miR397-laccase module in regulating expression of the abovementioned genes could not be established at present, though certain possibilities could be considered. Firstly, miR397-mediated downregulation of laccases may perturb cellular networks such as those related to cell wall modification, thereby indirectly altering expression of these genes. Secondly, some of the nine-downregulated genes may be direct targets of *Musa*-miR397 which are currently not predicted. Thirdly, other targets of *Musa*-miR397 may exist, which have not been assayed for in our study, but which may control these genes, leading to the observed changes. This work also uncovered possible regulation of CK signaling by the *Musa*-miR397-laccase module, but missing links remain to be identified. Future investigation is needed to expand our understanding of *Musa*-miR397-regulated plant growth.

### Overexpression of *musa*-mir397 does not compromise abiotic stress tolerance in banana

**Figure 4:**
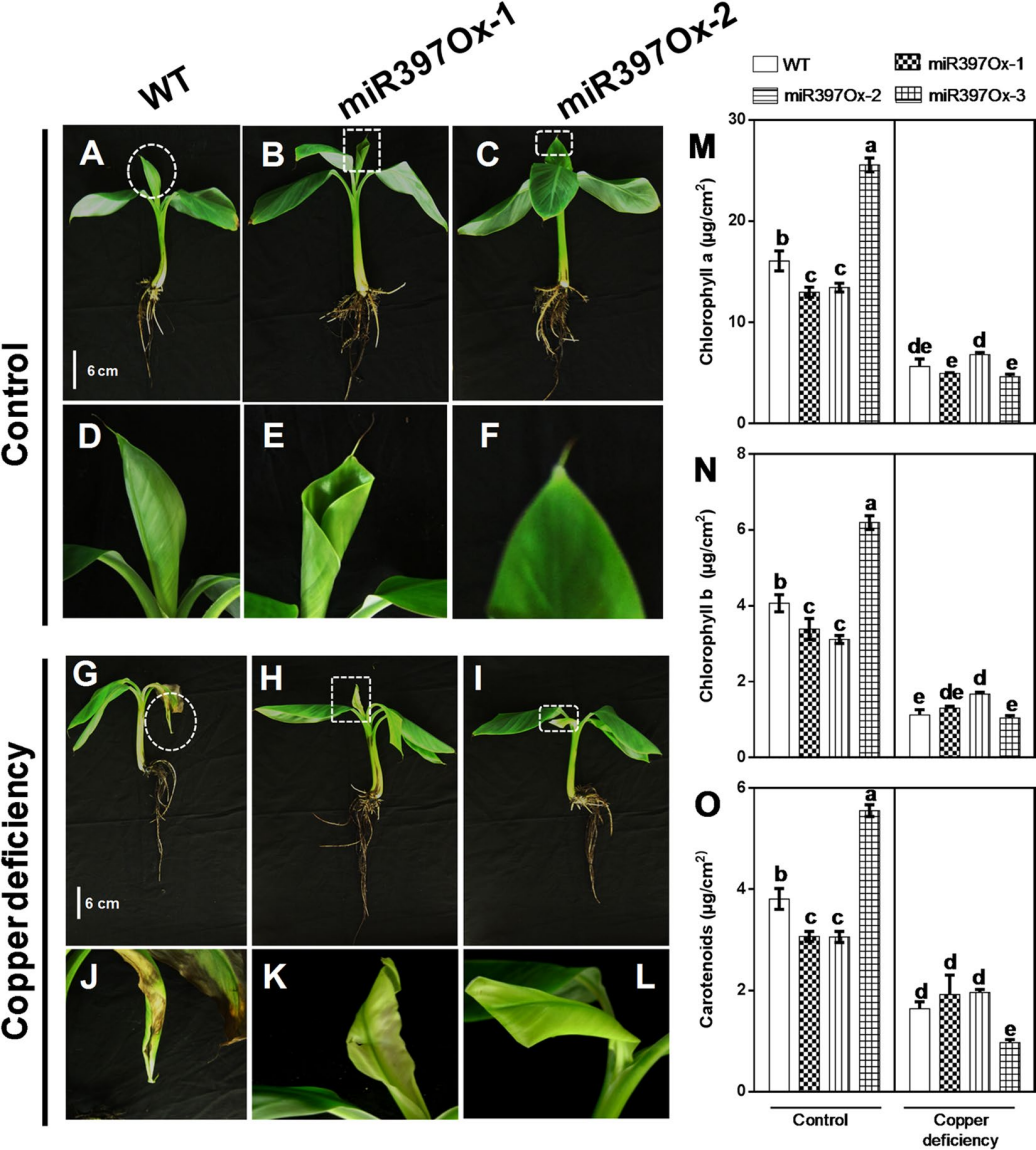
Copper deficiency stress tolerance assay for *Musa*-miR397Ox lines. Hydroponically grown *Musa*-miR397Ox lines and WT plants were subjected to copper deficient conditions (0 µM copper + 200 µM BCS) for a period of 2 mo. (**A**–**C**) WT, *Musa*-miR397Ox-1 and *Musa-*miR397Ox-2 lines respectively under control conditions. (**D**–**F**) Insets of the leaves from plants in (**A**–**C**), as enclosed by the dotted circle, square and rectangle respectively. (G-I) WT, *Musa-*miR397Ox-1 and *Musa-*miR397Ox-2 lines respectively under copper deficient conditions. (**J**–**L**) Insets of the newly emergent leaf from plants in (**A**–**C**), as enclosed by the dotted circle, square and rectangle respectively. Note the curling of the margins and severe chlorosis in the WT and transgenic plants under copper deficiency compared to the control. (**M–O**) Chlorophyll a, chlorophyll b and carotenoid content (ug/cm^2^) in WT plants and transgenic lines under control and copper deficient conditions. Bars represent the mean value of at least 3 plants ± SE. Means were compared using one-way ANOVA and Duncan’s multiple range test. Small alphabets represent statistically significant differences in the means.

As described earlier, miR397 is associated with ROS responsiveness in rice^94^. Considering that *Musa*-miR397 was significantly responsive to Cu deficiency and NaCl (Figs 1 and 2), we imposed Cu deficiency and salinity stresses as typical micronutrient and ionic stresses respectively, on wild-type and lines *Musa*-miR397Ox-1, *Musa*-miR397Ox-2 and *Musa*-miR397Ox-3. Both wild-type (Fig. 4G; inset in 4J) and *Musa*-miR397Ox-1 and *Musa*-miR397Ox-2 (Fig. 4H–I, insets in 4K–L) displayed similar phenotypes under Cu deficiency and control conditions. Under Cu deficiency, the youngest leaf initially developed whitish spots, which progressed to abnormal development with curled margins and severe chlorosis. This depicted the transition from mild (refer Fig. 1) to severe Cu deficiency, due to depletion of plant Cu reserves. In contrast, the control groups of both wild-type and *Musa*-miR397Ox lines unfurled healthy green leaves without morphological abnormalities (Fig. 4A, inset 4D and Fig. 4B,C, insets 4E,F). In view of observed chlorosis, and that Cu deficiency reduced chlorophyll content (Chl) in poplar^95^, we measured Chl a, b and carotenoid (CA) content in the youngest leaf from treated and control groups. All three parameters were significantly lower in Cu deficient plants compared to control condition and the decrease was similar in extent for both wild-type and transgenic plants (Fig. 4M–O). The observed phenotype indicates that in both wild-type and transgenic plants, severe Cu deficiency overrode the Cu-salvaging function of *Musa*-miR397.

**Figure 5:**
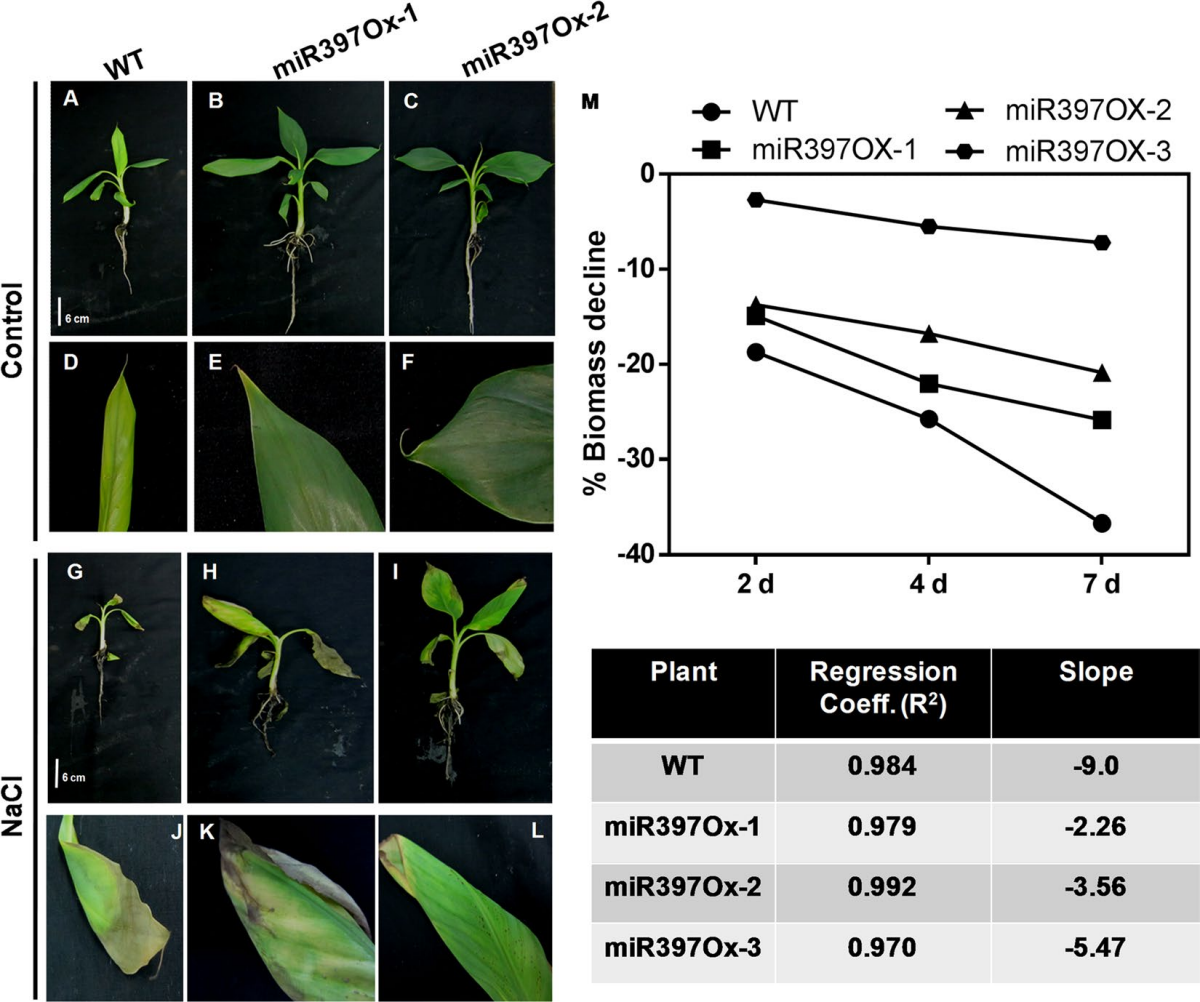
NaCl stress tolerance assay for *Musa*-miR397Ox lines. Hydroponically grown *Musa*-miR397Ox lines and WT plants were subjected to 90 mM NaCl for a period of 7 d. (**A**–**C**) WT, *Musa-*miR397Ox-1 and *Musa-*miR397Ox-2 lines respectively under control conditions. (**D**–**F**) Insets of the leaves from plants in (**A**–**C**). (**G**–**I**) *WT*, *Musa*-miR397Ox-1 and *Musa*-miR397Ox-2 lines respectively under 90 mM NaCl after 7 d of treatment. (**J**–**L**) Insets of the newly emergent leaf from plants in (**A**–**C**). Note the wilting of leaves and brown necrotic patches in the WT and transgenic plants under salt stress compared to the control. (**M**) Percentage decrease in biomass (FW) over 7 d in WT plants and transgenic lines under control and salt stress. The table shows the slope and regression coefficient of the linear trend lines set for the data shown in (**M**).

Agricultural soil salinity is widespread and responsible for serious crop losses of almost 50–80%^96^. We therefore challenged *Musa*-miR397 Ox lines and wild-type plants to salt stress. The wild-type (Fig. 5G; inset in 5J) and transgenic plants (Fig. 5H–I, insets in 5K,L) displayed typical symptoms of salt stress: leaf wilting and appearance of brown necrotic patches. Biomass (fresh weight; FW) declined over 7 days of treatment in both wild-type and transgenic lines. The decrease in FW was equivalent for both wild-type and transgenic plants over 4 days, but sharper for the wild-type between 4–7 days of stress, resulting in overall greater rate of decline in biomass for the wild-type (slope −9.0) than transgenic lines (slopes ranging from −2 to −5) (Fig. 5M). Under control conditions, both WT and miR397Ox plants had healthy growth and green leaves (Fig. 5A, inset 5D and Fig. 5B,C, insets 5E,F). Salinity stress strains the energy reserves of crop plants^97^ resulting in a balance between growth vigor and salt tolerance^98^. However, though *Musa*-miR397Ox plants had increased growth, they were not significantly compromised in stress tolerance relative to wild-type. In order to ascertain the basis for this phenotype, we turned to the RNA-seq data of the transgenic and wild type plants. Although the data were obtained under unstressed conditions, we observed that several stress-related genes such as *WRKYs*, the E3 ubiquitin ligases *PUB19* and F-box/kelch repeat protein, a chaperone protein *DNAJ8*, cytochrome b561 and an *ABCG40* transporter were upregulated in the transgenic lines relative to the wild type (Fig. 3C, Supplementary File S1).

Plant growth vigor and stress tolerance share a complex relationship. On one hand, trade-off between the two is observed for drought stress in *Arabidopsis*^99,100^, low temperature stress in *Nothofagus pumilio*^101^ and pathogen susceptibility in sunflower^102^. Contrastingly, mitochondrial *At*OXR2 enhanced both plant growth and resistance to ROS^103^, while activation of SA-mediated defense in *Arabidopsis* promoted seed yield and drought tolerance^104^. To the best of our knowledge, this is the first report in banana wherein overexpression of a native miRNA *Musa*-miR397 increased growth vigor without compromising tolerance to stress. This is desirable for banana production in unfavorable environments, because of rapid shrinkage of arable land. Our study also depicts remarkable conservation of miR397 function between *Arabidopsis*, rice and banana, indicating the essentiality of this miRNA in plant growth and development. Further investigation into the genes responsible for *Musa*-miR397-mediated growth enhancement will enrich our understanding of yield in this important staple fruit crop.

## Materials and Methods

### Computational prediction and characterization of *Musa-*miR397 and its targets in banana

Experimentally confirmed pre-miR397 sequences from different plant species were mined from miRBase and subjected to BLAST in the banana (*Musa acuminata*) genome database (https://banana-genome-hub.southgreen.fr). The consensus hit with maximum score and lowest E value was found to reside on chromosome 2 (GSMUA_Achr2T02982_001) and designated as *Musa*-miR397. This 116 bp sequence was analyzed in MFold (http://unafold.rna.albany.edu/?q = mfold) for its ability to fold into a stem-loop (Supplementary Fig. S3). Pre-*Musa*-miR397 was aligned with the pre-miR397 sequences from different plant species using CLUSTALΩ (https://www.ebi.ac.uk/Tools/msa/clustalo/) (Supplementary Fig. S3). The mature 21 nt *Musa-*miR397 sequence was then submitted to the psRNATarget server (http://plantgrn.noble.org/psRNATarget/)^105^ and queried against the *Musa acuminata* transcript database (JGI genomic project, Phytozome 12, 304_v1) for target prediction according to^106^. Among the predicted targets of miR397, seven laccase genes *Musa-Lac3T15320* (*Musa-Lac3*), *Musa*-*Lac11T24220* (*Musa*-*Lac11*), *Musa*-*Lac9T03350* (*Musa*-*Lac95*), *Musa-Lac9T03380* (*Musa-Lac98*), *Musa-Lac8T20780* (*Musa*-*Lac8*), *Musa-Lac6T34080* (*Musa*-*Lac6*) and *Musa-Lac2T16380* (*Musa*-*Lac2*) were chosen for further analysis (Supplementary Table T1). In all cases the miRNA was predicted to bind within the transcript coding sequence and cleave the mRNA. The target protein sequences were analyzed in the NCBI Conserved Domain Database to identify conserved motifs and the SignalP 4.1 server (http://www.cbs.dtu.dk/services/SignalP/) was used for secretory peptide prediction (Supplementary Fig. S6). *Musa*-miR397 upstream sequence analysis was carried out using the *cis*-element prediction tools PLACE^107^, PlantCARE^108^, and PlantPAN^109^ (Supplementary Fig. S1).

### Plant growth, physiological analysis and stress assays

Healthy *in-vitro* grown untransformed banana plantlets (wild type: WT) with profuse rooting were regenerated from banana embryogenic cell suspensions according to ref.^110^. Plantlets were acclimatized to half MS (pH 5.8) for 10 d in the growth chamber (Panasonic Healthcare Co. Ltd, Japan) at 25 °C, 70% relative humidity (RH) with a 16 h light/8 h dark photoperiod under fluorescent white light (40 W) till emergence of new leaf. Expression profiling of mature *Musa*-miR397 in wild-type plants was carried out under Cu deficiency, Fe deficiency and excess, salinity (NaCl), heat, methyl viologen (MV, 100 μM) and abscisic acid (ABA, 100 μM) treatment under hydroponic conditions. For Cu deficiency induction, plantlets were placed for 30 days in half MS medium (pH 5.8) with 0 μM Cu and 200 μM bathocuproine disulphonic acid (BCS). For Fe deficiency, plantlets were placed for 3 days in half MS without FeEDTA and supplemented with 300 μM ferrozine. For Fe excess treatment, plantlets were placed in half MS with 350 μM Fe for 3 days. Salinity stress was applied as 90 mM NaCl. Heat treatment was given by exposing plants to preheated growth chamber at 45 °C. Both MV and ABA were supplied in the nutrient medium and sprayed on the leaves. In all cases, wild-type plantlets exposed to half MS were taken as controls in each experiment for the respective time periods. Plants mock sprayed with sterile water were used as controls for ABA and MV treatments.

### Amplification, cloning of *Musa*miR397 and regeneration of transgenic plants

Primers were designed using the Primer3Web version 4.0.0 (http://primer3.ut.ee), to possess a Tm of 56 °C (Supplementary Table T2). Based on the sequence in the NCBI EST database (acc. no. FL666615.1), a 436 bp genomic sequence comprising the 116 bp putative pre- miR397 was amplified with primers ClFv and ClRv (Supplementary Table T2). This sequence was cloned into the pTZ57R/T vector and sub-cloned using *Pst1* and *Kpn1* enzymes into binary vector pCAMBIA 1301 under the *Zm*Ubi constitutive promoter and Nos 3’ terminator. After sequence verification, this construct (Supplementary Fig. S3) was electroporated into *Agrobacterium tumefaciens* strain EHA105 and used for banana transformation. Transformation, regeneration, greenhouse hardening and GUS histochemical staining protocols were followed as per^110^ (Supplementary Fig. S3). Transgenic plants generated were designated as *Musa*-miR397Ox lines. Six *Musa*-miR397Ox lines were hardened in the greenhouse and fresh leaf sample used for genomic DNA isolation as described previously^111^. PCR amplification of the *hygromycin phosphotransferase* II (*hptII*) gene was done using appropriate primers (Supplementary Table T2) to confirm presence of the T-DNA in the genome. Accordingly, a 788 bp product corresponding to the *hptII* gene was observed in the *Musa*-miR397Ox lines, but not in WT plants (Supplementary Fig. S3). PCR cycling was the same as described in^112^, except that the annealing temperature used was 56 °C for 1 min. The product was visualized on a 1% agarose gel. Southern blotting analysis was performed for selected lines to assess probability of stable transgene expression, as described previously^111,113^ (Supplementary Fig. S3). Selected transgenic lines were maintained in hydroponics in the same manner as wild-type plants. Both transgenic lines and WT plants were evaluated for growth performance, by measuring biomass (fresh weight) increase in hydroponics at chosen intervals. For stress assays on these transgenic lines, copper deficiency or salinity stress were applied similarly as in expression profiling for wild-type plants, except that treatment duration was 60 d for copper deficiency and 7 d for salinity stress. We measured chlorophyll content as per^114^.

### Mature miRNA detection by stem loop PCR

Total RNA from fresh leaf or root tissue was isolated using PureLink™ Plant RNA Reagent (Ambion, Invitrogen) according to manufacturer’s instructions. Genomic DNA was eliminated by DNase 1 (EN0525, Thermo Scientific) treatment, following manufacturer’s instructions. Approximately 70–80 ng of DNased RNA was reverse-transcribed using the stem loop primer designed against mature *Musa*-miR397 (Supplementary Table T2) as per the protocol of^115^. The cDNA was diluted 1:3 times and used for quantitative real time PCR for mature miR397 detection with SYBR Green Extract-N-Amp PCR ReadyMix (Sigma, USA) and the respective primers (Supplementary Table T2) according to manufacturer’s instructions (Supplementary Fig. S3). *Musa*U6 snoRNA was used as reference gene for normalization^116^. The mature miR397 sequence was confirmed after cloning the PCR products into the pTZ57R/T vector and sequencing.

### Quantitative real-time PCR

Total RNA was isolated from fresh leaf or root tissue and cDNA made according to^112^. The cDNA was diluted 10X and used for expression profiling of *Musa*-miR397 targets (Supplementary Fig. S3) and other genes. Primers were designed to flank the predicted miRNA-complementary sequence (Supplementary Table T2). PCR cycling conditions were as per^112^. The delta-delta Ct method of^117^ was employed to calculate absolute and fold (Log_2_) values of expression.

In all cases, at least 3 biological replicates were used and the experiment repeated at least twice.

### Estimation of micronutrient content in miR397Ox lines

Towards estimating the micronutrient content of miR397Ox plants over wild-type plants, inductively coupled plasma- optical emission spectrometry (ICP-OES) for Cu, zinc (Zn), Fe and manganese (Mn) was carried out on leaf samples following the method of^5^ (Supplementary Fig. S3).

### Histochemical staining for lignin deposition

Deposition of lignin in roots from a representative line *Musa*-miR397Ox-3 and WT plantwas visualized by staining with the polychromatic dye Toluidine Blue O as per^118^. Briefly, free-hand cut transverse sections of freshly harvested roots, were stained in a 0.5% (w/v) solution of Toluidine Blue O (Sigma, USA) in 2.5% Na_2_CO_3_buffer (pH 9.0), for 10 minutes. The sections were destained for 10 minutes and observed under light microscopy.

### Transcriptome analysis of *Musa*-miR397Ox lines and wild type plants

Towards elucidating the molecular basis for the growth phenotype of *Musa*-miR397Ox lines, we performed next-generation RNA-sequencing of two lines *Musa*-miR397Ox-2 and *Musa*-miR397Ox-3 along with wild-type plants, using two biological replicates. Two–month old hydroponically grown plants were selected and total RNA was isolated from third leaf from the top. RNA samples were sequenced on the Illumina HiSeq. 4000 platform by Genotypic Technology Pvt. Ltd (Bangalore) and clean reads obtained after adapter removal and filtering out of low quality bases. Reference-based mapping was carried out using the banana genome (https://banana-genome-hub.southgreen.fr). Differentially expressed genes (DEGs) obtained between the transgenic lines and wild type plants were filtered for significance according to adjusted p-value < 0.05 and analyzed further. RNA-seq data was deposited into GenBank with bioproject accession PRJNA543190 (experiment IDs: SRX5882018 - SRX5882023) which can be accessed at the URL: https://www.ncbi.nlm.nih.gov/bioproject/?term = 543190. Thirteen genes were selected for validation by RT-qPCR according to the delta-delta Ct method^117^ as described above (Supplementary Fig. S5).

### Phytohormone quantification by LC/MS

Hormone quantification from transgenic and WT plants was carried out by liquid chromatography/mass spectrometry (LC/MS) at the C-CAMP MS facility, Bengaluru. Briefly, frozen leaf samples in powder form, were extracted using 90% methanol, sonicated for 2 min and centrifuged for 5 min at maximum speed. The supernatant was transferred to a fresh tube and spiked with the highly polar internal standard Tryptamine-D4. The spiked samples were dried under vacuum and reconstituted in 50 µL of 20% methanol. Ten micro liters of this extract were injected for analysis. Standards for each phytohormone were serially diluted to prepare standard curves and processed in the same manner as samples. Instrument parameters are given in the supplementary Table T3.

### Accession numbers used in this study

The 116 nt sequence of *Musa* miR397 precursor miRNA was deposited into the NCBI GenBank with accession no. KT099188 and can be accessed at the URL: https://www.ncbi.nlm.nih.gov/nuccore/KT099188.1/.

**RNA sequencing data** was deposited into GenBank with bioproject accession PRJNA543190 (experiment IDs: SRX5882018 - SRX5882023) which can be accessed at the URL: https://www.ncbi.nlm.nih.gov/bioproject/?term = 543190. Thirteen genes were selected for validation by RT-qPCR according to the delta-delta Ct method^41^ as described above (Supplementary Fig. S5).

## Supplementary information

Supplementary information

Supplementary information

Supplementary information

## Data Availability

The datasets generated during the study were deposited in NCBI GenBank with the following URLs: https://www.ncbi.nlm.nih.gov/nuccore/KT099188.1/, https://www.ncbi.nlm.nih.gov/bioproject/?term=543190.
