# Supplementary material for: Overexpression of native Musa-miR397 enhances plant biomass without compromising abiotic stress tolerance in banana
Source: Sci Rep. 2019 Nov 11;9:16434. doi: 10.1038/s41598-019-52858-3 (PMC6848093; doi:10.1038/s41598-019-52858-3)
Supplement: Supplementary file 1 — Supplementary information [file 41598_2019_52858_MOESM1_ESM.pdf]

Overexpression of native *Musa*-miR397 enhances plant biomass without compromising abiotic stress tolerance in banana

Prashanti Patel[1], Karuna Yadav[1], Ashish Kumar Srivastava[2, 3], Penna Suprasanna[2, 3], Thumballi Ramabhatta Ganapathi[1, 3*]

[1]Plant Cell Culture Technology Section, [2]Plant Stress Physiology and Biotechnology Section, Nuclear Agriculture and Biotechnology Division, Bhabha Atomic Research Centre, Trombay, Mumbai, India; [3]Homi Bhabha National Institute, Mumbai, India.

E. mail addresses: prashantipatel12@gmail.com, karunayadavalt@gmail.com, ashishbarc@gmail.com, penna888@yahoo.com, **trgana@barc.gov.in**

***For correspondence trgana@barc.gov.in** (Thumballi Ramabhatta Ganapathi).

Telephone number: 022-25593276

SUPPLEMENTARY INFORMATION (FIGURES 1 - 6, TABLES 1-3)

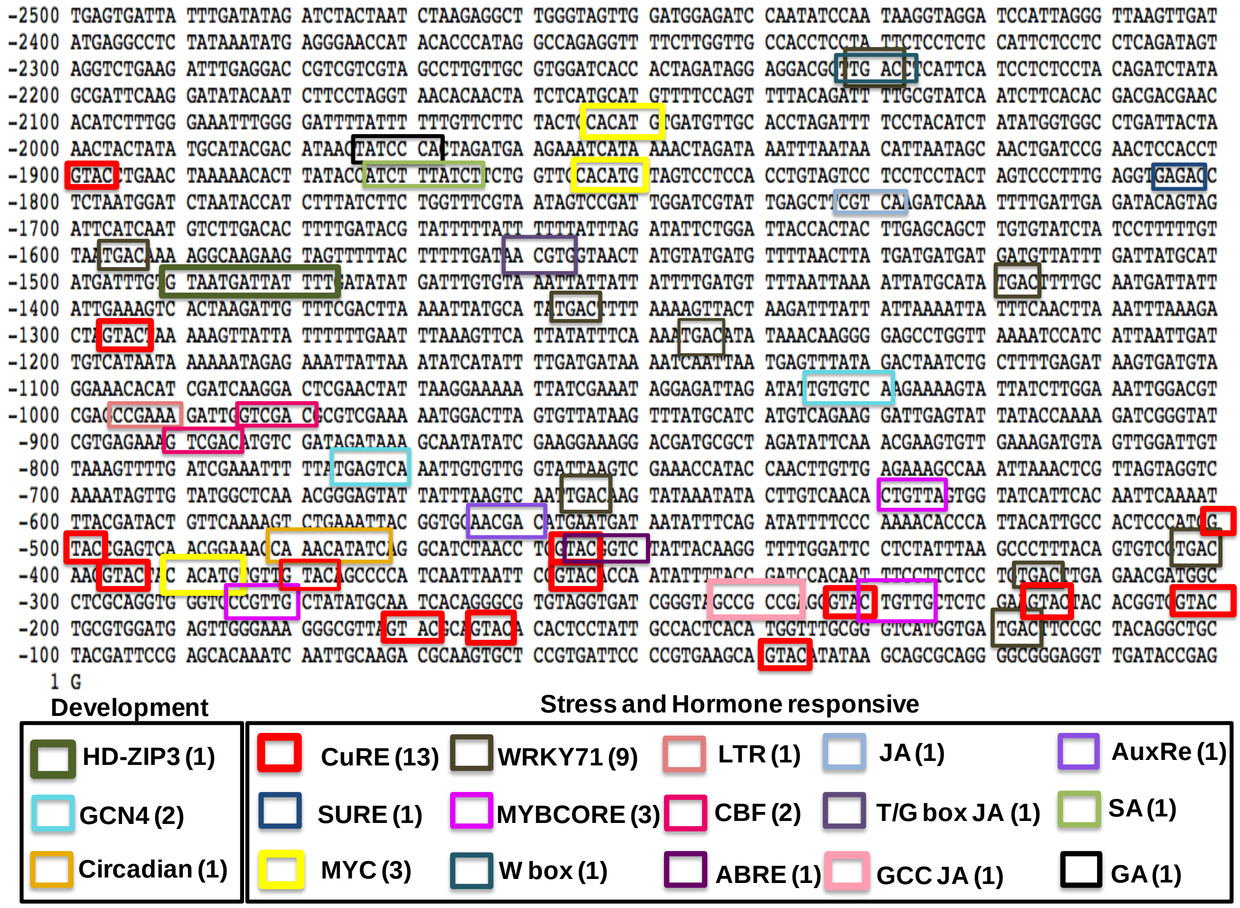

Supplementary Fig. S1. ***Cis*-element prediction in the upstream sequence of *Musa*-miR397**

A 2500 nucleotide sequence upstream of the pre-*Musa*-miR397 was chosen for analysis of *cis*-regulatory motifs associated with development, stress and response to hormones. Boxes indicate the motifs predicted by the PlantCARE, PLACE, and PlantPAN databases. Numbers in parentheses represent the copies of a particular motif in the sequence. Key: *CuRE*: copper- responsive element, *SURE*: sulfur- responsive element, GCN4: element for endosperm expression, CBF: C-repeat (CRT)/DRE (dehydration-responsive element) motif, LTR: low temperature responsive, ABRE: abscisic acid responsive element, JA: jasmonic acid, SA: salicylic acid, GA: gibberellic acid

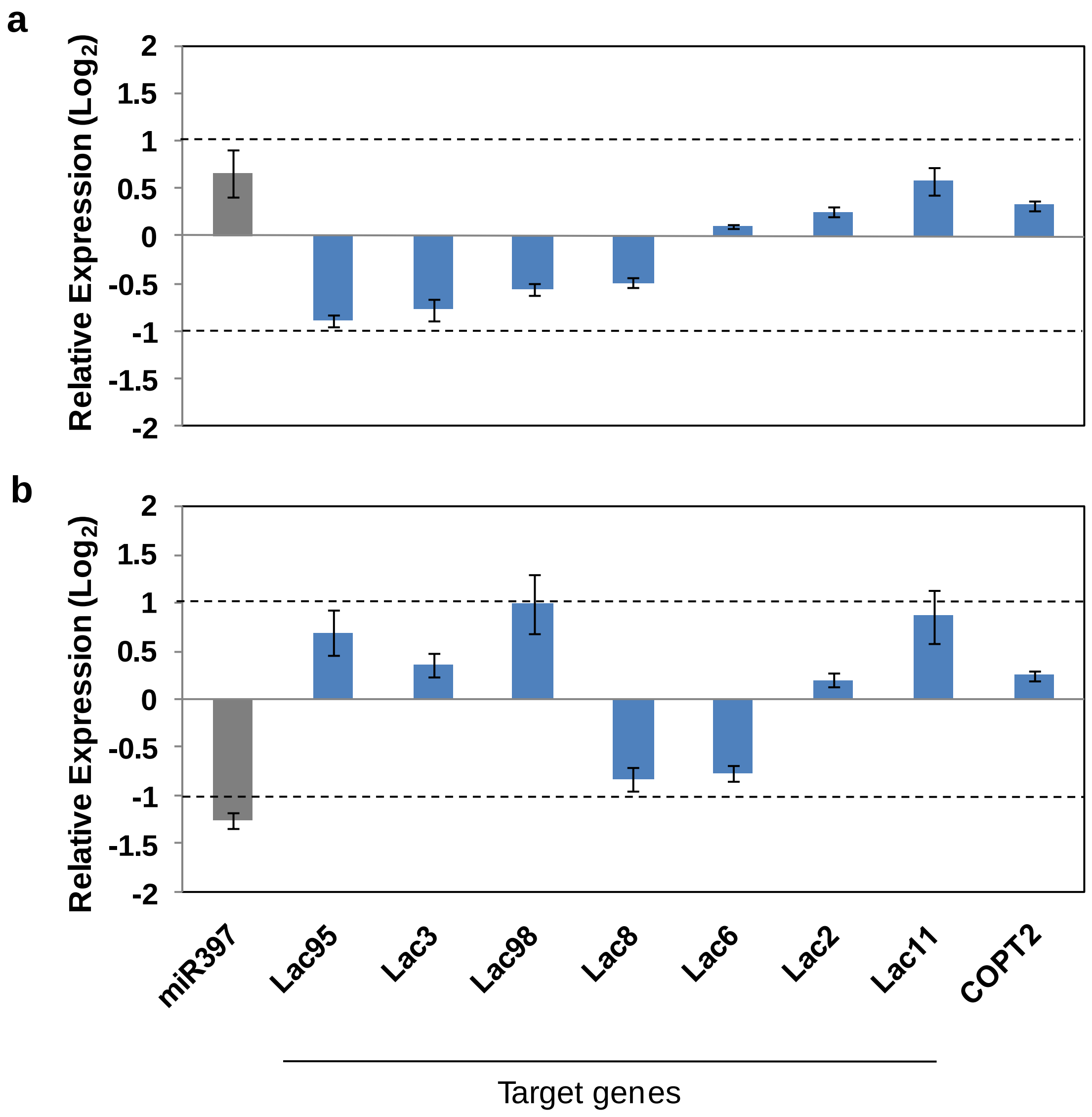

<u>Supplementary Fig. S2.</u> *Musa*-miR397-Laccase expression under iron stress conditions

Healthy hydroponically grown banana plantlets were challenged with iron deficiency (0 µM iron) or excess (400 µM iron). Plants growing in half strength MS with 50 µM iron were taken as controls. Expression profiling of *Musa*-miR397, target laccases (*Musa-Lac6*, *Musa-Lac2*, *Musa-Lac3*, *Musa-Lac11*, *Musa-Lac98*, *Musa-Lac8* and *Musa-Lac95)* and the root copper transporter *MusaCOPT2* was carried out under (a) iron deficiency and (b) iron excess conditions. Values are mean of 3 replicates ± SE and represent log_2 fold expression of the miRNA/target over control condition. Gene expression was normalized to that of *Musa-EFα*.Baseline represents expression of the genes in the control condition. Dotted lines indicate fold change cut-off of 1.

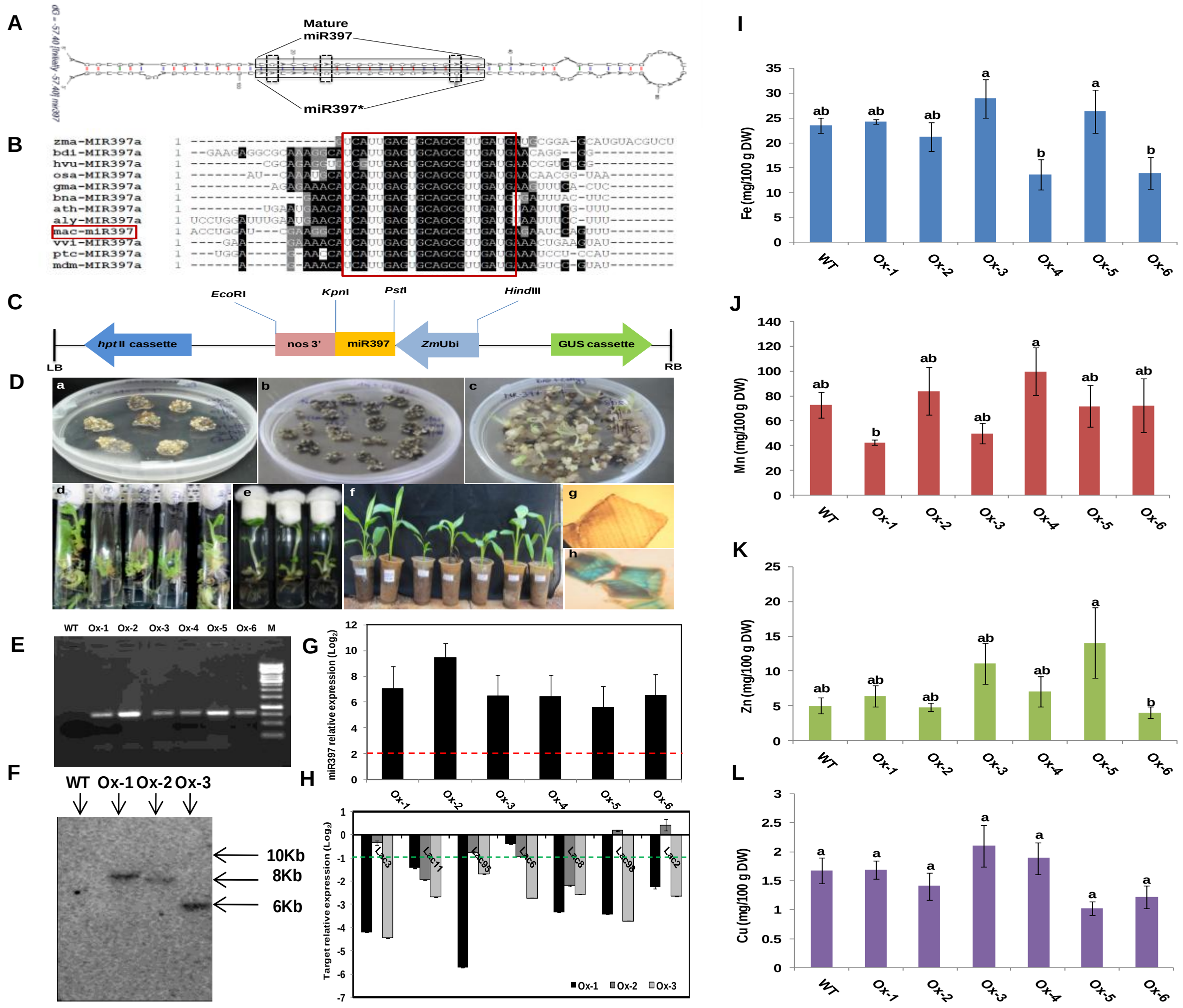

Supplementary Fig. S3. **Amplification, cloning and transformation of *Musa*-miR397 into banana** (A) Stem loop structure of 116 nt pre- *Musa*-miR397 isolated from the banana genome. (B) Alignment of pre- *Musa*-miR397 (mac-miR397) sequence with the pre-miR397 sequences from the following plant species: *Zea mays* (*zma*-miR397), *Brachipodiumdistachyon* (*bdi*-miR397), *Hordeumvulgare* (*hvu*-miR397), *Oryza sativa* (*osa*-miR397), *Glycine max* (*gma*-miR397), *Brassica napus* (*bna*-miR397), *Arabidopsis thaliana* (*ath*-miR397), *Arabidopsis lyrata* (*aly*-miR397), *Vitus vinifera* (*vvi*-miR397), *Populus trichocarpa* (*ptr*-miR397), *Malusdomestica* (*mdm*-miR397). The red box highlights the conserved 21 nt sequence of the mature miR397. (C) T-DNA region of the pCAMBIA 1301- miR397 binary vector representing the *Musa*-miR397 pre-miRNA sequence cloned under the constitutive *Zm*Ubi promoter andnos 3' terminator. (D) *Agrobacterium*-mediated transformation of the pCAMBIA 1301- miR397 vector into banana. (a) Formation of embryos in

bananaembryogenicmedium supplemented with 5 mg/L hygromycin. (b) Transfer of embryos to germination medium containing 0.5 mg/L benzylaminopurine (BAP) and supplemented with 5 mg/L hygromycin. (c) Regeneration of putatively transformed shoots on cytokine-medium containing 2 mg/L BAP + 30 mg/L adenine sulfate and supplemented with 5 mg/L hygromycin. (d) Multiple *in-vitro* clonal shoot production from putatively transformed shoots on cytokine-medium. (e) Rooting of individual plantlets on MS medium supplemented with 1 mg/L naphthalene acetic acid (NAA). (f) Two month-old greenhouse-hardened putatively transformed plants. (g-h) GUS histochemical staining of leaf tissue from putatively transformed (g) and wild-type plants showing intense blue coloration in transformed tissue. (E-H) Molecular characterization of *Musa*-miR397 overexpressing transgenic lines (*Musa*-miR397Ox lines 1-6). (E) Genomic PCR of *hptII* gene showing presence of 788 bp band in transformed plants. (F) Southern blotting for *hptII* gene in WT and transgenic lines *Musa*-miR397Ox-1, Ox-2 and Ox-3. Approximate band positions are according to a 10 kb DNA ladder. (G) Expression levels of mature miR397 in transgenic lines. (H) Expression levels of target laccases in three transgenic lines Ox-1, Ox-2, Ox-3 compared to WT. Values are mean of 3 replicates ± SE and represent $\log_2$ fold expression of the miRNA/target over WT. *Musa-U6* and Musa-*EFα* were used as reference genes for normalization in case of (G) and (H) respectively. Baseline represents expression of the genes in the control condition. (I-L) Profiling of micronutrient content in WT and transgenic lines (as mg/100g dry weight) for iron (Fe), manganese (Mn), zinc (Zn) and copper (Cu). Bars represent the mean value of atleast 3 replicate plants ± SE. For each element, means were compared using one-way ANOVA with Duncan's multiple range test. Small alphabets represent statistically significant differences in the means.

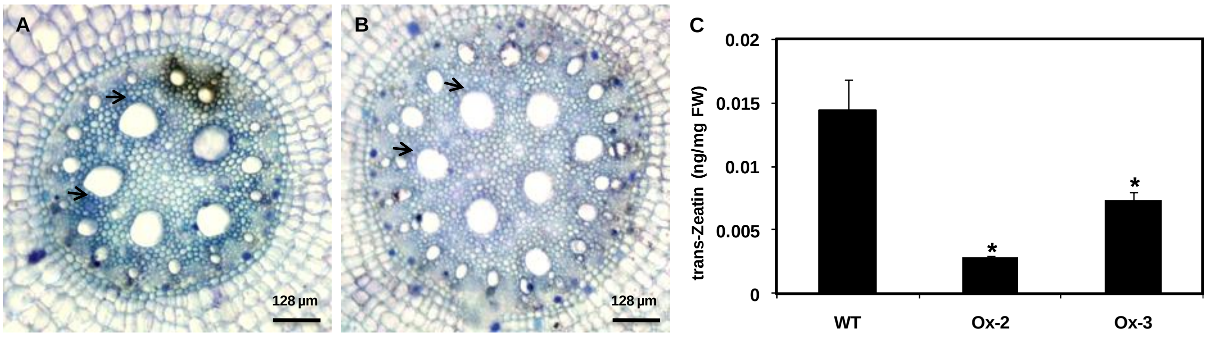

<u>Supplementary Figure S4.</u> Altered lignification and cytokinin content in *Musa*-miR397Ox lines

(A, B) Toluidine blue O staining of lignin in vascular bundles of root cross sections from transgenic and WT plants respectively. Arrows highlight stronger bluish-green staining around vascular bundles in (A) compared with *Musa*-miR397Ox-3 (B), indicating lower lignin deposition in the transgenic root. Scale bar = 128 µm. (C) Trans-zeatin levels (ng/mg fresh weight) in leaves of WT and two transgenic lines. Asterisks indicate significant difference measured by Student's t-test at a significance level of 0.05.

UPREGULATED GENES

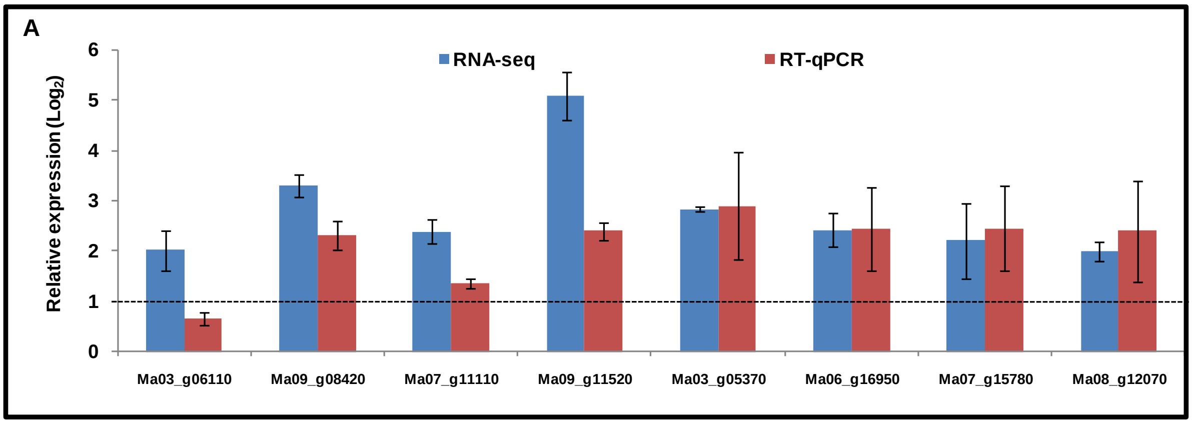

DOWNREGULATED GENES

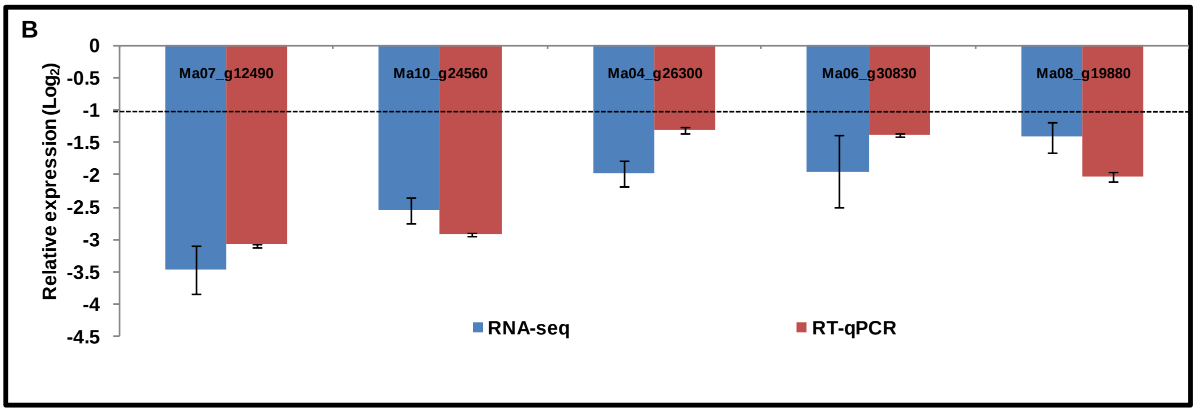

<u>Supplementary Fig. S5.</u> Validation of selected DEGs by RT-qPCR

In order to validate the expression data obtained from RNA-sequencing of the *Musa*-miR397Ox-2, *Musa*-miR397Ox-3 and WT plants, we carried out quantitative reverse-transcriptase PCR (RT-qPCR) of thirteen selected DEGs, taken from the list of 62 common up- and 9 common down-regulated genes in File S2. (A) Validation of 8 genes commonly upregulated between both transgenic lines and WT plants. (B) Validation of 5 genes commonly downregulated between both transgenic lines and WT plants. Bars in blue and red indicate data from RNA-sequencing and RT-qPCR respectively. For both RNA-seqand RT-qPCR data, values represent average of the $\log_2$ fold gene expression over WT, in two lines each with two biological replicates ± SE. For RT-qPCR, gene expression was normalized to that of *Musa-EFα*. Baseline represents expression of the genes in the control condition. Dotted lines indicate fold change cut-off of 1.

```
9T03380     1 --------MGGVRTFLLAACILFSS-----AQLSAGVTRHYKFDITLRNATRLCTTKSIV
6T34080     1 MGVV--SSAVPVRTLLLAATIFLAS-----AELSSGATRHYKFDITLRNVTRLCKTKSIV
2T16380     1 MGSFPCPSSVLVEALVVSSIVLLALPADVVQAQQNGIVRHYKFDIQMADVTRLCYTKSII
9T03350     1 MASSS--SDLLVKAFLLSCIALLALSDA-AVAPPTGITRHYKFDIQMANATRLCHTKSIV
8T20780     1 MESTVQHSMICSLAWLLALSTLLALPEL----SCGSTTRRYKFDIRLQNVTRLCRTRSIV
3T15320     1 MGSFHASIVIV----LLGFL-------MLVSVPTEAATKKYQFDVVVSNVSRLCHAKPVV
11T24220    1 MEP---CSWRA----LILVF-------FLLPLLTQGRTRHYKFNVVAKNFTRLCSPKSII
```

non-conserved signal peptide sequence

```
9T03380    48 TVNGQFPGPRIVVREGDRLVVKVVNHVRSDITIHWHGIRQVQSGWADGPEYITQCPIRTG
6T34080    54 TVNGEFPGPKIVAREGDRVVVKVVNHVENNITIHWHGIRQLRSGWADGPEHITQCAIQTG
2T16380    61 TVNGQYPGPTIFARDGDRVIVDVVNHVRDNVTIHWYGIRQLRSGWADGPAYVTQCPIQSG
9T03350    58 TVNGQFPGPKIVAREGDRVVVKVVNHVEHNVTIHWHGIRQLRSGWADGPAYVTQCPIQTG
8T20780    57 TVNGKFPGPKIMAREGDRVVVKVVNHVTNNVTIHWHGVRQLRTGWYDGPAYVTQCPIRTG
3T15320    50 TVNGRLPGPTIYAREGDRLIVNVTNYAQYNMTIHWHGVKQLRNGWADGPAYVTQCPIRSG
11T24220   47 TVNGRFPGPTLYAREGDTVIVKVANHVQENVTIHWHGVRQLTTGWADGPAYVTQCPIQPG
```

CuBD

```
9T03380   108 QSYVYNFTTAGQRGTLWWHAHASWLRATVYGSLVILPKRGVPYPFARPYKEVPIILGEWF
6T34080   114 RSYVYNFTLAGQRGTFWWHAHASWLRATVYGALIILPKPGVPYPFPKPYKEVPIIFGEWF
2T16380   121 QSYVYNFTIVGQRGTLFWHAHISWLRTTLHGAIIILPELGVPYPFIEPYKEVPVIFGEWW
9T03350   118 HSYVYNFTIVGQRGTFFWHAHISWLRATLYGPIVVLPQLGVPYPFAKPYKEVPVILGEWW
8T20780   117 QSFVYNFTIVGQRGTLFWHAHISWNRATLYGPIVILPKRGVPYPFPKPYKEIPVIFGEWF
3T15320   110 GSYTYHFNVTGQRGTLWWHAHILWLRATVHGAIVINPEQGVPYPFPQPYQETELILGEWW
11T24220  107 QNYAYNFTLTGQRGTLLWHAHIMWQRATVHGAIVILPKRGVPYPFPAPHKEVVLVLAEWW
```

CuBD

```
9T03380   168 NADPEAVIAQALRTGGGPNVSDAYTINGLPGPLYNCSG--KDTFKLKVKPGKTYLLRLIN
6T34080   174 NADPEAVIAQALQTGAGPNVSDAYTINGLPGRLYNCSG--KDTFKLKVKPGKTYLLRLIN
2T16380   181 KADTEVVISQALENGGEPNISDAFTINGLPGPLYNCSV--KDTFKLMVSPGKSYLLRLIN
9T03350   178 KADAEAVISQALETGGGPNVSDAYTINGLPGPFYNCSA--KDTFKLKVKPGTTYLLRLIN
8T20780   177 NVDPEAIIAQALRTGAGPNISDAYTINGLPGPLYNCSK--KDTFELKVKPGKTYLLRMIN
3T15320   170 NADVEKVEQQGNLLGLPPNTSDAHTINGKPGPLFPCPE--KHTYALEVESGKTYLLRIIN
11T24220  167 KSDVEAVINEALQSGLGPNVSDAHTINGHAGPTSGCASSARDGFTLRVDQRKTYLLRIIN

9T03380   226 AALNDELFFGIANHTVTVVETDASYVKPFDAEALVITPGQTANGTFDNTTTAGVLEYHNP
6T34080   232 AALNDELFFSIANHTVTVVETDANYVKPFDADTVLVTPGQTTNVLLHAKPTFPNAAFLFA
2T16380   239 AALNDELFFSIANHTVTVVEVDAGYVKPFEADTILISPGQTMNVLLDAKPNYPNAIFSMS
9T03350   236 AALNDELFFGIANHTLTVVDVDAVYVKPFESDALLISPGQTTDVLLRAKPDYPNAMFFMS
8T20780   235 AALNDELFFSIANHTLTVVDVDAVYVKPFEADIVHIAPGQTTNVLLHTKPSPPDATFLMA
3T15320   228 AALNDDLFFAIAGHSMTVVEIDATYCKPFNTSALVIAPGQTTNVLVRADQAP--GRYFMA
11T24220  227 AALNDDLFFKVAGHLMTVVEVDATYTKPFTIDTLLIAPGQTTNVLLTADQGA--GRYLVT

9T03380   286 NRA-------------------------------STKLPLLRPALPSLNDTANVTNFASK
6T34080   292 AR--------------------------------PYATGQGTPALPSLNDTAYATNFSSK
2T16380   299 ARPYSTGS-GAFDNTTVAAIL---------------------------------------
9T03350   296 ASPYVTGS-GTFDNSTVAGVLEYRNPNSSSRASFNSKLPLYKPTLPSLNDTSFFANFTGK
8T20780   295 ARPYATG-----------------------------------PTLPALNDTAYTTNYTGK
3T15320   286 TRSFMDIPIPV-DNKTATAILQYRGVP--------TTVVPVMPQLPAPNDTSFVASFSDQ
11T24220  285 ASPFMDSPLVAVDNSTGTATVQ--------------------------------------

9T03380   315 LRSLADDRYPSDVPRTVDRRFYFTIGLGTDPCPTNRTCQGPNGTKFAASVNNASFVFPST
6T34080   320 LRSLADARYPANVPRTVDQRFYFTVGLGADPCPANQTCQGPSGTKFAASINNVSFAFPST
```

```
2T16380    319 --NLATAQYPANVPQTVDRRFFFTVGLGTDPCPTNQTCQGPNGTKFSSTVNNISFTEPTT
9T03350    355 LRSLATARFPANVPQAVDRRVFFTVGLGSSPCPENQMCQGPNGTKFSATVNNVSFALPTT
8T20780    320 LRSLASAQFPANVPKTVERRFYFTVGLGTSPCAKNQTCQGPNGTKFAASVNNISFALPTS
3T15320    337 LRSLNSPRYPASVPLDVDRRLFYTIGLGVNPCPTC-----LNGTRLTASLNNITFVLPQV
11T24220   307 ---YTDAQYPANVPSTVDHSLLFTVGLGVNPCPAC-----TNGSRVVAAMNNVTFMMPTT

9T03380    375 -ALLQAHYYSQSNGVYTTDFPDIPPFPFNYTGTPPNNTFVSKGTKVVVLPFNTSVELVLQ
6T34080    380 -ALLQAHYLARSYGVYTTDFPNNPPSPFNYTGTAPNNTFVSHGTKVVVLPFNTSVEVVMQ
2T16380    377 -ALLQAHFFGQSEGVYTPDFPAFPLMPFDYTGTPPNNTNVSHGTKLLVLPFNTSVELVMQ
9T03350    415 -ALLQAHFLGQSRGL-------------------------------MVLPFNTSVELVMQ
8T20780    380 AALLQAHYFGQTKGV-------------------------------VVLPFNTSVELVMQ
3T15320    392 -ALLQAHYYDM-KGVI---------------------------------AFNSTVELVLQ
11T24220   359 -ALLQAHYFNM-SGVFTNDFPGQPLIAFNYTGSGPNNTQTMNGTRLYRLPYNASVQLVLQ

9T03380    434 DTSIQGIENHPMHFHGHNFHVVGQGFGNYDPVTDPCNFNLVDPVEKNTAGVPVGGWLAIR
6T34080    439 DTSILGAENHPMHLHGHNFYVVGTGFGNYDPMNDPPKFNLVDPVEKNTVGVPAGGWLAIR
2T16380    436 DTSILGTESHPLHLHGYNFFVVGEGFGNFDPASDPAEFNLVDPVERNTVGVPDGGWVAIR
9T03350    443 DTSILGAESHPLHLHGFNFFVVGQGFGNYDPAKDPTKFNLVDPVERNTVGVPAGGWVAIR
8T20780    409 DTSIQGAESHPLHLHGFNFFVVGTGFGNYDPSKDPRRFNLADPIERNTVGVPVGGWVAIR
3T15320    417 DTNLLMVESHPFHLHGYNFFVVGTGIGNFDPAKDPETFNLIDPPERNTVGVPTGGWTAIR
11T24220   417 DTGIIGPESHPIHLHGYNFFVVGRGVGNYDPATSPSKFNLVDPIERNTMAVPAAGWTAIR
CuBD

9T03380    494 FRAINPGAWLNHCHIDLHLSWGLKMVWVVNDGPLPNQKLPPPPSDLPRC-----------
6T34080    499 FLAINPGAWLNHCHIDVHLSWGLRMAWLVNDGALPTQKLPPPPSDLPRC-----------
2T16380    496 FLAINPGVWFNHCHIEIHLSWGLTMAWLVLDGALPNQKLPPPPSDLPKC-----------
9T03350    503 FLAINPGVWFNHCHLEVHTSWGLKMAWLVLDGSLPHQKLPPPPSDLPKC-----------
8T20780    469 FLAINPGVWFNHCHLEVHTSWGLRMAWVVNDGWLPNQKLVPPPSDLPRC-----------
3T15320    477 FRAINPGVWFNHCHLEVHTSWGLKMAFVVENGDGPEESILPPPMDLPPC-----------
11T24220   477 FRAINPGVWFIHCHFEVHTTWGLKMAFVVEDGKGPNESLQPPPKDLPASSCGPAHDPRKP
CuBD
                                                         P rich C
                                                         terminal

9T03380        ------------------------------------------------------------
6T34080        ------------------------------------------------------------
2T16380        ------------------------------------------------------------
9T03350        ------------------------------------------------------------
8T20780        ------------------------------------------------------------
3T15320        ------------------------------------------------------------
11T24220   537 YFSLSVPPFLPFRLHRRDHRVLRSKLPAPPRRRLTQSPLLRCRPPLSLGNYLLAELKKNI

9T03380        ------------------------------------------------------------
6T34080        ------------------------------------------------------------
2T16380        ------------------------------------------------------------
9T03350        ------------------------------------------------------------
8T20780        ------------------------------------------------------------
3T15320        ------------------------------------------------------------
11T24220   597 LPVEVDGPCGWISASCRFQYYENISIYTPDKWVHLPKIMQPKTGIPYSCIPFVYQFAFWF

9T03380        -----
6T34080        -----
2T16380        -----
9T03350        -----
8T20780        -----
3T15320        -----
11T24220   657 MFRQI
```

<u>Supplementary Fig. S6.</u> Conserved motifs in target laccase protein sequences

The predicted protein sequences of 7 target laccases *Musa*-Lac98 (9t03380). *Musa*-Lac6 (6t34080), *Musa*-Lac95 (9t03350), *Musa*-Lac8 (8t020780), *Musa*-Lac2 (2t16380), *Musa*-Lac3 (3t15320) and *Musa*-Lac11 (11t24220) were aligned using CLUSTALΩ. Several conserved motifs depicted by colored boxes are shown. The large red box at the N- terminal region depicts the non-conserved signal peptide sequence in the laccase proteins. Key: *CuBD*: copper ion-binding domain; *P-rich C terminal*: Proline residue rich C-terminal.

SUPPLEMENTARY TABLES (1-3)

Target Acc.	E - value	UPE	miR start	miR end	Target start	Target end	miR alignment sequence	Target alignment sequence	Inhibition type
3T15320	1.5	12.109	1	21	675	695	UCAUUGAGUGCAGCGUUGAUG	CAUCAACGCUGCCCUCAAUGA	Cleavage
9T03350	1.5	12.860	1	21	699	719	UCAUUGAGUGCAGCGUUGAUG	CAUCAACGCUGCACUCAACGA	Cleavage
11T24220	1.5	18.492	1	21	672	692	UCAUUGAGUGCAGCGUUGAUG	CAUCAACGCGGCACUCAAUGA	Cleavage
9T03380	1.5	12.386	1	21	669	689	UCAUUGAGUGCAGCGUUGAUG	GAUCAACGCUGCACUCAACGA	Cleavage
2T16380	1.5	12.669	1	21	708	728	UCAUUGAGUGCAGCGUUGAUG	GAUCAACGCUGCACUCAACGA	Cleavage
6T34080	1.5	12.411	1	21	687	707	UCAUUGAGUGCAGCGUUGAUG	GAUCAACGCUGCACUCAACGA	Cleavage
8T20780	1.5	17.32	1	21	696	716	UCAUUGAGUGCAGCGUUGAUG	GAUCAACGCUGCACUCAACGA	Cleavage

Supplementary Table T1. *Musa*-miR397 target prediction

The mature sequence of *Musa*-miR397 was analyzed in the psRNAtarget server to predict putative target mRNA sequences from the *Musa acuminata* transcript database (JGI genomic project, Phytozome 12, 304_v1). The parameters were as follows: Maximum expectation value (E-value): 2.0; Target accessibility (UPE): 25; flanking length around target site for UPE analysis: 17 bp in upstream/13 bp in downstream; range of central mismatch leading to translational inhibition: 9–11 nt

Supplementary Table T2. Primers used in this study

Gene	Forward Primer (Fv)	Reverse Primer (Rv)
**Amplification primers 5’-------->3’**		
Hygromycin phosphotransferase	GTCCTGCGGGTAAATAGCTG	ATTTGTGTACGCCCGACAGT
miR397 gene cloning primers (Cl)	AGGCCTCGAAGCAACAAGAAC	GTTTGCGGGTCATGGTGATG
**Reverse Transcriptase-quantitative PCR (RT-qPCR) primers**		
Elongation factor EF-α	CCGATTGTGCTGTCCTCATT	TTGGCACGAAAGGAATCTTCT
pri- Musa-miR397	TTGAGTGCAGCGTTGATGAG	CGTCGAAGGCATTATTGGGT
mature-miR397 stem loop Fv	GCGGCGGTCATTGAGTGCAGCG	-
Universal reverse	-	GTGCAGGGTCCGAGGT
miR397 stem loop primer	GTCGTATCCAGTGCAGGGTCCGAGGTATTCGCACTGGATACGACCATCAA	
Musa-Lac3	CCTCCCAACACTTCGGATGCTC	CGTTTGACCTGGTGCAATGACC
Musa-Lac11	GTGGAAATCGGACGTTGAAGCC	AAGAGGTCGTCATTGAGTGCCG
Musa-Lac95	TCCTTTCTACAATTGCTCCGCT	ATTAGGGTAATCAGGCTTGGCG
Musa-Lac6	CTTCCCGGGCGCTTGTATAATTG	GTGCAAAAGGACGTTGGTGGTC
Musa-Lac8	CTCCCTGGCCCCTTGTACAATT	GAGGACTAGGCTTGGTGTGGAG
Musa-Lac98	GGCCAAATGTTTCCGACGCTTA	GGGGTGATAACGAGGGCTTCAG
Musa-Lac2	GGAAAGTCGTACCTCCTTCGCT	CGGATCCAGTAGAGTAGGGCCT
Musa-COPT1	AGACGCACTACACCCACATG	CGAAGAGCAGGAACCCGAG
Musa-COPT2	GGGCAAGAACTCCGAGATCC	CGAGCTGCCGAAGAGAAGAA
Musa-COPT5	ACAGATTCTTGGGCGGGTTA	TGGCGAGCATGAGGAGGTAT

Musa-COPT10	ACTCGGCATGTACCTCCTC	ATTGGATTCAGCTCGTCGGA
Musa-FRO2-1	GGCATCCTCCAGCGTTACTA	TCCGCATTGTAGAACCAGGA
Musa-FRO2-2	CGATGAGAAGGAACGAACCC	GTGGCTGTTGAAGTGGAAGA
Musa-FRO2-3	CTCTGCTCGTTGGCCATTG	CACACACGAAGACACCCACT
Musa-FRO24	CCTCTTTTGTCGCATTCCTCG	AACCAGAAACCGGGCGAA
Musa-FRO25	TAGTCTGGCTGCATGGAAGA	AGAGGTCCCCAAGTAATGCC
Musa-FRO26	TCACCATGCTCGATCTCCTC	GGAATGCGACGAAAGAGGAC
Musa-YSL6	GCAGTGCCTTTCTACATTGGA	CAGATTGGGGGATCAATCCTA
Musa-YSL8	CATGGCCATACCGTTCTACC	GGCGTCTACCTTGTCGTTGT
Musa-YSL12	CCATCGACATGTGTGTAGGC	CTCATTTCCACACACGGATG
Musa-ZIP7	CCTTCGCCATATCAAGCCCC	TATCACTCCAACCCCTGCGA
Musa-ZIP8	GTGGACTATCAGCCTGCACA	ATGAAGACCCCAGTTGCGAG
Musa-OPT4	CCTGCAACCCCAACCAACAT	ATGCCAACGGACAGTGATCG
Musa-OPT6	GTGTGGGTGTTGAGCAAGGT	ACGCCCATGAATGCTGTACC
Musa-OPT10	CCATGGCGGGCTGTATAGGA	ATGGTCCCTGTGATGAGCCA
**Genes used for RNA-seq validation**		
**Ma09_g08420**	ACGGTTCACCTTCGACTCCT	GCCTTGCAGATGACAGACCA
**Ma03_g05370**	TTTGGAGAGAAGAGAATGCGAGT	GCCTATGTCGAACAATGCCTTC
**Ma06_g16950**	GAGATGTCCGAACTAGAGTCCAC	CGTCGTCTGCATTGGTTCCA
**Ma07_g11110**	GGCTGCGGTGGTTCAATCAA	GAGCCATGATGACGTGCCAG
**Ma09_g11520**	GCGAGACTGGGAAAGCAGTT	ATGCGTGATGAACTCCCCAC
**Ma07_g15780**	AGCTTGATGTCCCAAAGAGCC	AGCACCCTATACCTGCCTCTA
**Ma03_g06110**	CGGGAAGATACCTCAGGCGA	AAGAACACCCACTGCATCGC
**Ma09_g12070**	GAGGTCAACAACGCATCATGG	TGAGCAGAGGTCTTGGGGTAT
**Ma01_g24560**	CCAAACTCAGCCTCGGGAAA	AGTTCCCCACCCAGACGTAG
**Ma04_g26300**	GCGATGCAAAGGAGGGGAAG	TCCCAGTTACACCCACCGTT
**Ma06_g30830**	TGTCGAAGCTGGTCCTGAGA	TGCCCCTCGTTTGATCCTCT
**Ma08_g19880**	GATGAACAAGATGAGCGGCC	CACCTCGCCATTTCTCGACA
**Ma07_g12490**	TCTACCTCATCCTGCACCAC	TCGTGGTTGTATGGCGAATG

Supplementary Table T3. Instrument Parameters for LC/MS

LC set-up

**Instrument**	**Agilent 1290 Infinity UHPLC**
**Column**	**AgilentEclipse Plus, 1.8µ, C18, 50mmx2.1mm**
**Mobile Phase A**	**10mM Ammonium Acetate in Water (0.1%FA)**
**Mobile Phase B**	**Acetonitrile (0.1%FA)**
**Flow Rate**	**0.2ml/min**
**Column Oven**	**40°C**
**Auto-sampler Temp**	**10°C**
**Injection volume**	**10µl**
**Run Time**	**30mins**
**Gradient**	**0-2mins:5%B, 2-19mins:5-55%B,19-20mins:55-95%B, 20-25mins:95%B, 25-25.1mins:95-5%B, 25.1-30mins:5%B**

MS set-up

**Instrument**	**Thermo Fisher-TSQ Vantage**
**Spray Voltage (+ve)**	**4000V**
**Spray Voltage (-ve)**	**2500V**
**Vaporizer temp**	**200°C**
**Sheath gas flow rate**	**30Arb**
**Aux gas flow rate**	**10Arb**
**Peak width settings**	**0.70FWHM**
**Scan time**	**0.05s**
**Injector settings**	**0-2.5mins:waste, 2.5-16mins:load, 16-30mins:waste**
